# Hypothalamic wars: the last nanodelivery

**DOI:** 10.1007/s11154-026-10022-z

**Published:** 2026-03-04

**Authors:** Sajid Fazal, Jane Jose Vattathara, Miguel López

**Affiliations:** 1 NeurObesity Group, Department of Physiology, CIMUS, University of Santiago de Compostela, Santiago de Compostela, 15782 Spain; 2 CIBER Fisiopatología de la Obesidad y Nutrición (CIBEROBN), Santiago de Compostela, 15706 Spain

## Abstract

The hypothalamus plays important roles in maintaining overall body homeostasis and energy balance. Disruption of its normal functioning has been associated with the onset of various metabolic disorders in the body that arise from several genetic, immunological, and environmental factors or their combination. To combat this, developing treatment strategies that modulate the activity of the hypothalamus could be advantageous. Here, understanding the hypothalamus’s complex structure and function within the context of regulation of energy balance is important to identify treatment targets and to explore opportunities to modulate key metabolic pathways. Moreover, the unique sensitive position of the hypothalamus in the brain necessitates that treatment strategies that are developed are highly effective and specific, and do not cause any untoward effects at neighboring regions of the brain. This is further complicated by its protection through the blood-brain barrier (BBB) that highly regulates the entry of materials from the periphery, allowing entry of molecules only under specific conditions. In this regard, advanced multifunctional nanoparticulate drug-delivery systems can be of benefit as they have been explored for brain specific delivery. For this, nanoparticle chemistry, specific ligand expression, BBB penetration capability, biocompatibility, and immunogenicity among other physicochemical properties are important parameters that govern brain specific delivery. In this review, the role of hypothalamus in regulating energy metabolism, its structure and function and the effect of dysfunction on the onset of metabolic disorders are summarized. Furthermore, the use of nanoparticles for brain targeting and hypothalamic regulation, such as small extracellular vesicles targeting AMP-activated protein kinase (AMPK), along with the future of nanoparticle-based modulation of the hypothalamus is also discussed.

## Introduction

Currently, the global prevalence of metabolic diseases is on the rise [1–4], which continues to be a major public health challenge [1–4]. Type-2 diabetes, hypertension, obesity, hypercholesterolemia, and metabolic dysfunction-associated fatty liver disease are the most common metabolic disorders [1–4]. Among them, the prevalence of obesity has increased at an alarming rate with studies showing that the total number of children, adolescents, and adults living with obesity worldwide has surpassed one billion and that there are more obese people now than underweight [5–12]. 

**Fig. 1:**
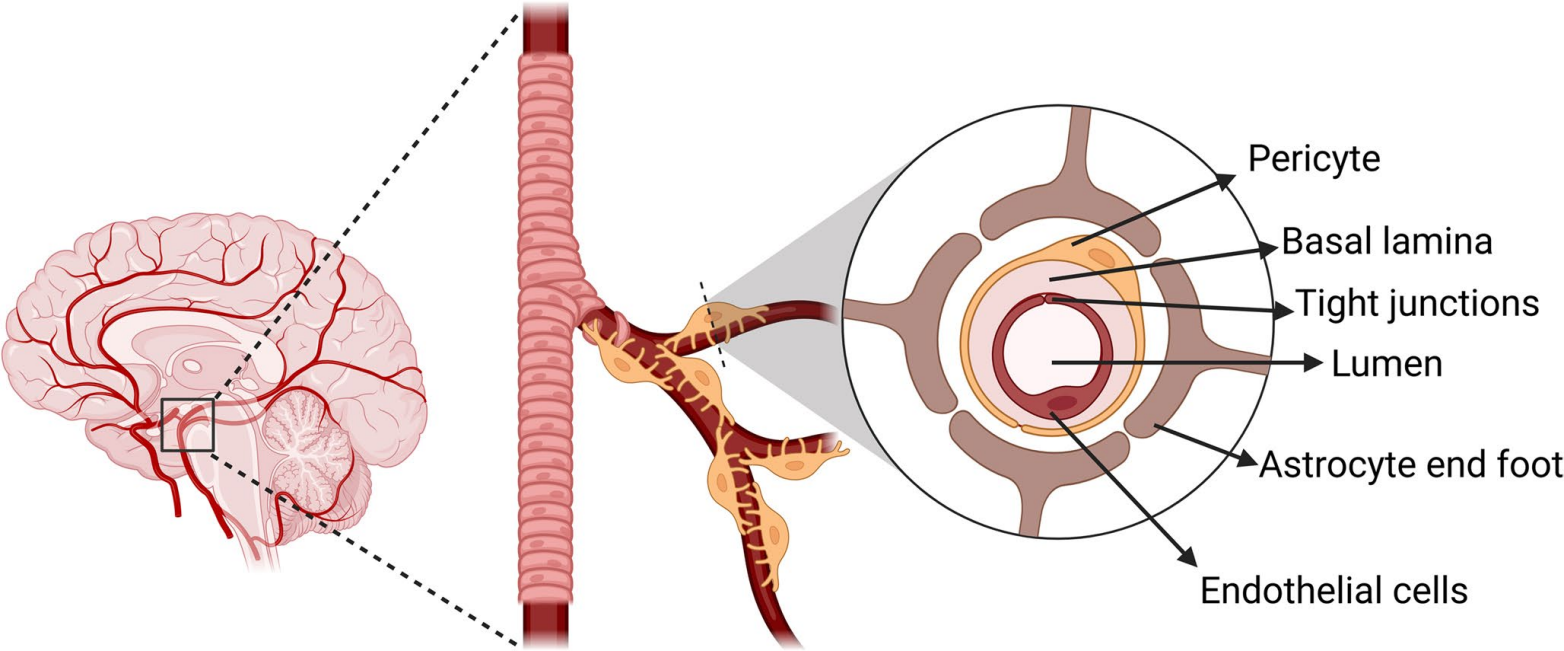
Structural organization of the blood–brain barrier. Schematic representation of the blood–brain barrier (BBB) at the level of a brain microvessel. The luminal side is formed by a continuous monolayer of endothelial cells sealed by tight and adherens junctions, which strongly restrict paracellular diffusion and contribute to the high transendothelial electrical resistance characteristic of the BBB. These endothelial cells rest on a basal lamina that also surrounds pericytes, which closely interact with the endothelium to regulate barrier integrity, vessel stability and transport properties. On the abluminal side, astrocytic end‑feet almost completely ensheathe the vascular wall, providing metabolic and structural support, while interacting with neurons and microglia to couple local blood flow and nutrient supply to neuronal activity. Together, these specialized cellular and extracellular components create a highly selective interface that tightly controls the entry of ions, nutrients, hormones and xenobiotics from the bloodstream into the brain parenchyma, thereby preserving central nervous system homeostasis

Obesity has long been considered to be caused by external environmental conditions, that could be prevented through lifestyle changes and regular exercise. However current understanding of the disease pathology of obesity has unearthed its underlying genetic factors and the complex involvement of the central nervous system in its exacerbation which makes its treatment complicated [3, 7–15]. Since obesity is a condition pertaining to energy imbalance, dysregulation in the body’s central metabolic regulator, namely the hypothalamus, plays a major function in its onset and progression [13, 16–21]. The hypothalamus is located in the ventral part of the brain above the pituitary gland and surrounding the third ventricle. Just like all other parts of the brain, the hypothalamus is also protected by the blood-brain barrier (BBB) [22–30] (Fig. 1). The positioning of the hypothalamus within the brain permits direct exposure to various hormonal and nutritional signals through its distally positioned part, namely the median eminence (ME). The ME is a circumventricular organ having highly fenestrated capillaries that allow signaling molecules from the periphery to reach different regions of the hypothalamus [22–30]. The ME comprises axon terminals of hypothalamic neurons and the capillary network of hypophyseal portal system placed in apposition with the pituitary gland and serves as a link between the brain and the endocrine system [22–30].

The dysfunction in the individual hypothalamic nuclei brought about by pro-inflammatory conditions, genetic factors and impaired hormone signaling aggravate the condition of not only obesity, but also other metabolic diseases as well [13, 16–22]. In this regard, devising therapeutic strategies that can alter/rectify these aberrations will be useful in the treatment of associated metabolic diseases [13, 19, 20]. However, due to the extremely complex and highly heterogeneous functions of the hypothalamic neuronal populations [13, 16, 17, 31–33], it is difficult to target a single neuronal population with a drug to obtain a desired effect. Additionally, the BBB further makes accessing the hypothalamus from the periphery difficult since it highly regulates biomolecule entry into the brain [13, 20, 23–30, 34]. Thus, a successful therapeutic modality should not only be able to penetrate the BBB but also be able to target specific hypothalamic nuclei to modulate neuro-metabolic pathways [13, 20, 23–30, 34].

In the recent past, nanoparticles have demonstrated successful brain penetration and drug delivery [20, 35, 36]. Owing to their small size, in vivo stability, modifiable surface functionality, biodegradability, and capability to protect encapsulated drug cargo from external environmental conditions, nanoparticles have revolutionized the field of medicine and drug-delivery [20, 36–42]. For brain-specific delivery, nanoparticles have shown exceptional capability to penetrate the BBB (via intravenous administration) [24, 29, 43–45] or the mucosal barrier (intranasal administration) [46, 47] and have shown promising results for the treatment of different disease conditions such as stroke [45, 48], glioblastoma [49] and other neurological conditions such as Alzheimer’s [50], Parkinson’s [51] diseases etc. Moreover, studies have shown their ability to target specific sites within the brain either through the use of targeting ligands [52–54], or through their inherent capability to home to these sites (e.g. using extracellular vesicles [20, 36, 43–45, 55–57], cell-membrane nanovesicles [58]). Due to these promising studies reported in literature, and the vast amount of modularity on offer to engineer nanomaterials, it is highly envisaged that nanoparticles could be the next precise silver bullet for the treatment of metabolic disorders through brain targeting, specifically the hypothalamus [20, 36].

## Role of the hypothalamus in regulating metabolism

The hypothalamus is a vital part of the brain conferred with the control of many autonomic nervous functions with a strong association with the endocrine system due to its interactions with the pituitary gland [13, 16–22, 59, 60]. The hypothalamus is therefore considered as a neuroendocrine entity not limited to regulating energy balance but also in regulating circadian rhythms, reproductive activity, emotional and behavioral patterns [13, 16–22, 59, 60]. To do so, the hypothalamus produces neurotransmitters, neuropeptides, and hormones for executing multiple functions [13, 16–22, 59, 60]. Shouldering such immense complex functions makes the hypothalamus segmented with each region possessing well-defined hypothalamic neuronal clusters or nuclei [13, 16–22, 59, 60].

The hypothalamus is a highly metabolically responsive area of the brain playing crucial role in the regulation of energy homeostasis by the combinatorial actions of its different nuclei [13, 16–22]. The main hypothalamic nuclei that regulate energy homeostasis comprises the arcuate nucleus (ARC), the paraventricular nucleus (PVH), the ventromedial nucleus (VMH), the dorsomedial nucleus (DMH) and the lateral hypothalamic area (LHA) [13, 16–22]. These together regulate energy balance by controlling feeding (homeostatic and hedonic), energy expenditure (physical activity and thermogenesis) and whole-body metabolism [13, 16–22, 61]. All these regulations for maintaining energy homeostasis are brought about by the actions of these nuclei by tuning the expression of neuropeptides in response to metabolic hormones like insulin, ghrelin, leptin, adiponectin, glucagon like peptide (GLP-1), estrogens, thyroid hormones (THs) and energy currencies (glucose, lipids and amino acids) from the periphery [13, 16–22, 59, 60, 62]. Thus, the hypothalamus integrates metabolic signals from the periphery and responds efficiently to the energy needs. Dysfunction or inflammation in key neuronal and glial populations within those nuclei contributes to obesity, insulin resistance, type 2 diabetes, and metabolic syndrome [13, 16–22]. Along with this, genome-wide association studies (GWAS) have recently determined genes in hypothalamic neuronal populations that can specifically tune energy balance, and their expression levels are identified as predisposing factors for obesity and other metabolic disorders [7, 13]. The targetability of these areas remains mainly unaddressed [13, 20, 36]. Furthermore, these identified genes are found to be altered significantly in populations of high body mass index, thus emphasizing its importance [7]. This was corroborated with the established pathophysiology of metabolic diseases (gathered over many years) comprising of hypothalamic inflammation, autophagic dysfunction, mitochondrial damage, neuronal apoptosis and structural abnormalities of the human hypothalamus [13, 16–22, 63]. Moreover, due to the extremely complex and highly heterogeneous functions of the hypothalamic neuronal populations, it is difficult to target a single neuronal population with a drug to obtain a desired effect [13, 20, 36]. Rather, it would be more promising to use a multi-receptor or combinatorial drug administration regime to modulate hypothalamic function effectively such as the complex mechanism of action of the recently approved GLP-1 derived drugs (liraglutide, semaglutide and tirzepatide [8, 11, 14, 15].

## Hypothalamic dysfunction in obesity

Obesity is a condition driven by prolonged excessive nutrient uptake exacerbated by a sedentary lifestyle which leads to reduced energy expenditure and abnormal increase in adiposity and body weight (BMI > 30) [5–12]. This condition has long been considered to be caused by the direct effect of external environmental conditions, that could be ameliorated through lifestyle changes and regular exercise [8, 10–12]. Though increased physical activity and a calorie deficit diet would help in preventing the onset of obesity, its treatment on the other hand is complicated [8, 10–12]. This reflects the current understanding of the pathophysiology of obese conditions and the underlying genetic conditions that may play a role in its exacerbation.

One of the important monogenic causes of obesity is the mutation in the melanocortin receptor 4 gene (MCR-4) [7, 13, 64, 65]. This gene is widely expressed in the PVH encoding a G-protein coupled receptor whose natural ligand is alpha-melanocyte stimulating hormone (α-MSH; a proteolytic product of proopiomelanocortin, POMC) [66–69]. This mutation is widely reported in obese conditions and other associated metabolic comorbidities in both humans and rodent models [7, 13, 64, 65]. Similar to monogenic causes there are other polygenic mutations and genetic polymorphisms that are identified in obesity [7, 13, 65], such as leptin receptor mutations (resulting in abrogated anorexigenic response [7, 13, 70]), heterozygous single-minded ***(****SIM1)* gene mutations (causing PVH abnormalities leading to hyperphagic obesity [7, 13, 71, 72]), *PCSK1* gene mutations (encoding for the prohormone convertase 1/3, a pro-neuropeptide converting serine endoprotease [7, 13, 73]) and the *FTO* (fat mass and obesity associated gene) a gene encoding for 2-oxoglutarate-dependent nucleic acid demethylase [7, 74–76]. These are a few among the widely reported gene clusters that show single nucleotide polymorphisms (SNP) and mutations that result in weight gain [7, 65].

Apart from the genetic predispositions cited for obesity, hypothalamic inflammation is another facet that contributes to the progression of an obese phenotype [13, 21, 63, 77–81]. Consumption of high fat diet (HFD) triggers hypothalamic inflammation upon the release of chemokines and cytokines ensuing an inflammatory scenario in the brain [63, 77, 82, 83]. This central inflammation could possibly cause neuronal apoptosis in association with microgliosis and astrogliosis which abrogates the tight regulation imparted by the hypothalamic neurons in energy homeostasis [63, 79, 84, 85]. Furthermore, hypothalamic inflammation activates key signal transduction pathways *via* the NF-κB and stress responsive kinase c-Jun N terminal kinase (JNK) [63, 82, 86–88]. The activation of these deleterious signaling pathways causes further deterioration of neuronal health by activating unfolded protein response (UPR) pathway eliciting endoplasmic reticulum (ER) stress [78, 82, 88–94]. This inflammatory chaos further compromises the integrity of the BBB, facilitating the recruitment of peripheral inflammatory mediators into the CNS [30, 95, 96]. Some of these changes occurring within the hypothalamus of experimental models of obesity are also well documented in humans using multi-modal neuroimaging studies [80, 97]. The degree of hypothalamic inflammatory markers and gliotic markers are now considered as predictive tools for insulin sensitivity, body weight gain and the likelihood of the incidence of associated co-morbidities [13, 21, 28, 63]. In addition to these mechanisms, defects in the Bardet–Biedl syndrome (BBS) protein complex (the BBSome) have emerged as a cause of hypothalamic leptin resistance and severe obesity [98–101]. It has been shown that BBSome components are required for proper trafficking of the long signaling form of the leptin receptor (LRb) to the plasma membrane in hypothalamic neurons, and that disruption of BBS proteins impairs LRb surface expression and signaling, leading to obesity in mouse models [102, 103]. This work highlights intracellular trafficking machinery such as the BBSome as an additional layer of regulation in hypothalamic energy‑balance circuits, beyond classic inflammatory or ER‑stress pathways.

Methods to modulate hypothalamic activity hold great promise for the treatment of various metabolic and endocrine disorders that stem from its dysfunction. However, such methods to modulate hypothalamic function remain largely unexplored [20, 36, 104–108]. This likely reflects the highly complex role that the hypothalamus plays in maintaining body homeostasis, and the difficulty associated in targeting due to its sensitive location and structural complexity. Additionally, the BBB further makes accessing the hypothalamus from the periphery difficult. In this regard, it is important to utilize a multifunctional therapeutic that can not only modulate specific neuro-metabolic pathways within the hypothalamic nuclei but also be able to specifically target the hypothalamus and undertake successful entry to the brain though the BBB. Such a multifunctional therapeutic, in essence must be a composite material that can assimilate multiple components in a single platform that can work together to achieve a desired therapeutic outcome. In this regard, the field of nanoscience and nanotechnology has played a decisive and much needed advancement in the field of pharmaceutical and medical science.

## Nanoparticles for brain delivery

The use of nanoparticles (NPs) as protective delivery vehicles for active pharmaceutical ingredients (APIs; such as drugs, small molecules, proteins, peptides, nucleic acids, etc.) has led to the development of the now well-known field of nano-drug-delivery [37–42]. This approach of utilizing NPs for cargo delivery provides unique advantages over the use of the bare API alone [37, 40, 109, 110] by **(i)** preventing direct exposure to degrading enzymes (such as hydrolases, lysozymes, etc.) that may chemically alter the API, **(ii)** modulating immune system interactions of APIs that may otherwise be immunogenic [111], **(iii)** chemically presenting stealth/non-immunogenic reactive groups on NP surface [112, 113] (such as polyethylene glycol, poly sarcosines, etc.) that will improve its biodistribution and circulation times and **(iv)** presenting actively targetable functional moieties that will provide specificity and organ/tissue tropism [114, 115]. All these in-effects will reduce undesirable degradation and clearance of the API of interest and overall increase its pharmacokinetic and pharmacodynamic properties [116, 117]. Moreover, importantly, in cases where the API of interest can produce undesirable toxic effects (for e.g., chemotherapeutics used for cancer therapy), the shielding effect provided by nanoparticles can alleviate its non-specific systemic toxicity and improve its overall safety. Furthermore, in certain cases, depending on the type of material used, nanoparticles can also be utilized for remote-controlled on-demand drug delivery upon exposure to an external stimulus [118, 119], like magnetic fields [120], light [121–123], ultrasound [124, 125], pH [126, 127], that would specifically elicit a response enabling the release of encapsulated cargo. Due to all these advantages conferred, nanoparticles have now become important tools for drug-delivery. This is further supported by the fact that many current approved formulations in the market are nanoparticle based [128].

Several reports have highlighted the use of nanoparticles for brain-specific delivery. As with any exogenous material administered in the body, when utilizing nanoparticles for drug delivery applications, several intrinsic factors can influence its bio-distribution profile. These include its physicochemical characteristics such as size, charge, chemical make-up, reactive functional groups, bio-degradability, hydrophilicity, etc., which will in turn modulate its interaction with biological fluids, serum proteins/biomolecules, and cells of the immune system [129–131]. Here, the route of administration plays an obvious, yet important role in the type of environment the nanoparticles are exposed to initially. For delivery to the brain, two main modes of nanoparticle administration have been widely reported in literature: intravenous [24] and intranasal [46, 47]. Unlike direct intrathecal delivery to the brain, these procedures are easy to implement, have high patient compliance, and do not require complicated high-risk surgical methods. In certain cases, though, a direct intrathecal delivery becomes necessary especially when the direct effect of the nanomaterial on the brain is to be evaluated [132–135]. In this review, however, we focus on brain/hypothalamus delivery through the intravenous and intranasal route as these modes of administration are considered desirable for easy clinical translation.

### Intravenous delivery for brain targeting

For brain delivery, the intravenous route of administration is a simple and effective mode of API administration. As with all APIs administered intravenously, nanoparticles also undergo systemic circulation and non-specific accumulation at different organs in the body including the lung, liver, spleen, and kidneys [129, 136]. Here, the % injected nanoparticle dose that accumulates at the brain has been reported to be between 0.01 and 0.5%), and has been found to be dependent on the type of nanoparticle administered, the integrity of the BBB, as well as on the use of targeting agents (coupled onto the nanoparticle surface) that can specifically target the BBB [137–140]. Among these, the BBB plays the most important role as it presents a major barrier to the entry of molecules from systemic circulation to the brain [23–25].

The BBB, which is located within the brain microvasculature **(**Fig. 1**)**, shares a general organizational plan with arterioles in the systemic circulation, but displays specialized structural and functional features that tightly regulate molecular entry into the brain [23–27]. However, it possesses certain key differences owing to the vital role and sensitivity of the brain. The luminal side of the arteriole in the BBB comprises of a single layer of endothelial cells that are tightly tethered to each other and are resting on the basal lamina. Any gaps between the endothelial cells are packed with tight junctions and adherence junctions, unlike the arterioles in systemic circulation which ensures that biomolecules that enter the brain are highly regulated and tightly controlled [23–27]. The transendothelial electrical resistance (TEER; which is a measure of the ionic permeability of these junctions) is about 100 to 500 times more than that of non-cerebral capillaries [141]. Within the basal lamina reside pericytes, which interact closely with endothelial cells and play key roles in modulating biomolecule entry and preserving BBB integrity [23–27]. On the abluminal side of the basal lamina astrocytic end-feet, neural synapses and microglia are present which make up the core components of the different parts of the brain. Here, the astrocytes act as an interface between the neural cells and the endothelial cells, besides serving important functions for the proper functioning of the CNS [23–28].

The BBB in the median eminence of the hypothalamus exhibits distinctive features, including increased endothelial permeability and reduced expression of tight junction proteins, and is closely associated with specialized ependymal cells known as tanycytes, which contribute to regulating the exchange of signals between the periphery and hypothalamic neurons [23–28, 142]. Additionally, capillaries of the ME harbor a wider perivascular space between the endothelial cell and the surrounding tissues [26–28]. The proteins that are expressed and released by the cells of the BBB dictate its permeability, for e.g., the increased expression of vascular endothelial growth factor (VEGF), matrix metalloproteinases (MMP’s), proinflammatory cytokines (IL-6, TNF-α), chemokines, nitric oxide etc. can destabilize the BBB and result in endothelial apoptosis and increased BBB permeability [26–28]. On the other hand, proteins like angiopoetin-1, glial cell derived neurotrophic factor (GDNF) and upregulation of tight junction proteins claudin and occludin can decrease BBB permeability [26–28]. Thus, structural plasticity of the cells comprising the ME will determine the rate and type of molecules entering and leaving the hypothalamus [143]. This leakiness of the BBB of ME is maintained to communicate rapidly to the periphery and respond to feeding cues and the overall metabolic status. This has been demonstrated through radiolabeled and fluorescent tracing studies that show the ability of systemically administered hormones to enter the ARC after trafficking the BBB in the ME and choroid plexus [144]. Hormones like leptin, insulin, GLP-1, prolactin etc. that have large molecular weights bind to their receptors on the ARC neurons, astrocytes and tanycytes [103, 142, 145]. These hormones and molecules like glucose and fatty acids are found to be internalized by the transporters on the glial end feet [103, 142, 145], while ghrelin (3 kDa) that is smaller in size is reported to passively diffuse the vasculature of the ME [146].

To enter the brain, nanoparticles will need to traverse through the BBB via the transcellular pathway (entry through cells), rather than the paracellular pathway (in between cells) since the tightly packed structures within the BBB (especially the tight and adherence junctions in the luminal side of the basal lamina) make paracellular transport highly unlikely for nanoparticles [23, 24, 29]. For transcellular entry into the brain, receptor mediated transcytosis is considered the most rational approach to delivery, wherein receptors on epithelial cells (such as transferrin receptor [147], insulin receptor [148–150], low-density lipoprotein receptors [151–154]) are exploited to uptake nanoparticles that present themselves with specific recognizable ligands. This recognition, followed by clathrin-dependent or independent endocytosis processes, leads to nanoparticle uptake through vacuoles, their fusion to early endosomes, and their release through vesicle fusion to the abluminal side of the basal lamina, resulting in the entry of nanoparticles to the brain [23, 24, 29].

Nanoparticle entry into the brain through receptor mediated transcytosis has been reported by utilizing different types of surface functionalized ligands. By utilizing Angiopep-2, a ligand having specificity to bind to low density lipoprotein receptor-related protein (LRP) in the BBB epithelium, ultra-small ceria nanoparticles (~ 4 nm) were found to enter the brain upon intravenous administration [155]. Even though non-specific biodistribution of these nanoparticles could be observed in the major organs such as the liver and spleen, their specific accumulation (µg nanoparticle/g of tissue), was found to be higher in the brain than in organs such as kidneys, lungs and heart [155]. Owing to their ROS scavenging capability, ceria nanoparticles were demonstrated here for the treatment of stroke. Thus, by targeting the BBB, entry to the brain was made possible in this case. Similarly, nanoparticles functionalized with ligands that recognize endothelial receptors such as transferrin [156] or insulin [157], or with integrin‑binding peptides such as the Arg‑Gly‑Asp (RGD) motif, which is recognized by several endothelial integrins expressed at the BBB [158], its entry into the brain can be strategically promoted .

Brain targeting can also be enabled by utilizing ligands for targeting receptors that are specific to the diseased condition, or by utilizing a characteristic property of the target site itself. For example, in the case of ischemic brain tissues, C-X-C motif chemokine receptor 4 (CXCR4) is significantly over-expressed [159], and the inclusion of such receptor specific ligands on the surface of nanoparticles can enable better targeting outcomes. This was demonstrated using polymeric nanoparticles that were conjugated with AMD3100 that binds to CXCR4 [160]. Furthermore, these polymeric nanoparticles consisted of an ROS reactive poly(2,2′-thiodiethylene 3,3′-thiodipropionate) (PTT) polymer, that could alleviate ROS stress [160]. The inclusion of AMD3100 as a targeting ligand, an ROS scavenging polymer, and the encapsulation of glyburide (used for the treatment of cerebral oedema), enabled important brain penetration upon intravenous administration which further improved stroke recovery in a stroke model of mice [159]. This shows how tuning nanoparticle design based on the characteristics of the diseased site can be helpful in enhancing targetability and cargo delivery. Such an approach was further demonstrated for the treatment of glioblastoma multiforme, a rapidly growing tumor, where its high metabolic activity and characteristic rapid nutrient uptake was taken into advantage [161]. For this, biomimetic nanoparticles made of albumin proteins were utilized since these tumor cells showed high albumin uptake due to the presence of albumin binding proteins SPARC (secreted protein acidic and rich in cysteine) and glycoprotein 60 (gp60) [162]. To further increase uptake in vivo, these nanoparticles were additionally functionalized with a cell penetrating peptide LWMP (low molecular weight protamine) that facilitated intracellular entry. Intravenous administration of chemotherapeutic drug loaded nanoparticles in this case, lead to significant reduction of tumor volume as compared to free drug controls [162].

By utilizing such BBB targeted nanoparticles, it is evident that specific brain accumulation is made possible. The binding affinity and avidity of the ligand to the brain endothelial cell receptor has been found to play an additional important role for nanoparticle entry into the brain parenchyma [156]. Although it may seem intuitive that nanoparticles tagged with high‑affinity, high‑avidity ligands would show superior BBB targeting, efficient receptor‑mediated transcytosis into the brain parenchyma instead requires an optimal, intermediate ligand avidity [156]. This is because an excessively high ligand avidity promotes nanoparticle retention on the luminal side of the BBB, whereas an avidity that is too low prevents efficient receptor binding altogether. This behavior was clearly demonstrated for transferrin‑conjugated gold nanoparticles, where increased accumulation in the brain parenchyma was observed only when an intermediate density of transferrin ligands was presented on the nanoparticle surface [156].

### Intranasal delivery for brain targeting

Compared to intravenous administration, intranasal delivery has several benefits, including enhanced brain bioavailability, reduced systemic exposure (and thus fewer systemic adverse effects), and a rapid onset of therapeutic effects [47]. The biggest advantage this route of administration offers is the bypassing of the BBB [163], which could make brain delivery relatively simple for drugs that have poor BBB penetrability [163]. In addition, from the applicability point of view, intranasal administration carries low injury risk and superior patient compliance owing to its ability to be self-administrable [47]. All these advantages underscore its importance, thus making this a favorable route of administration for clinical translation [164].

**Fig. 2:**
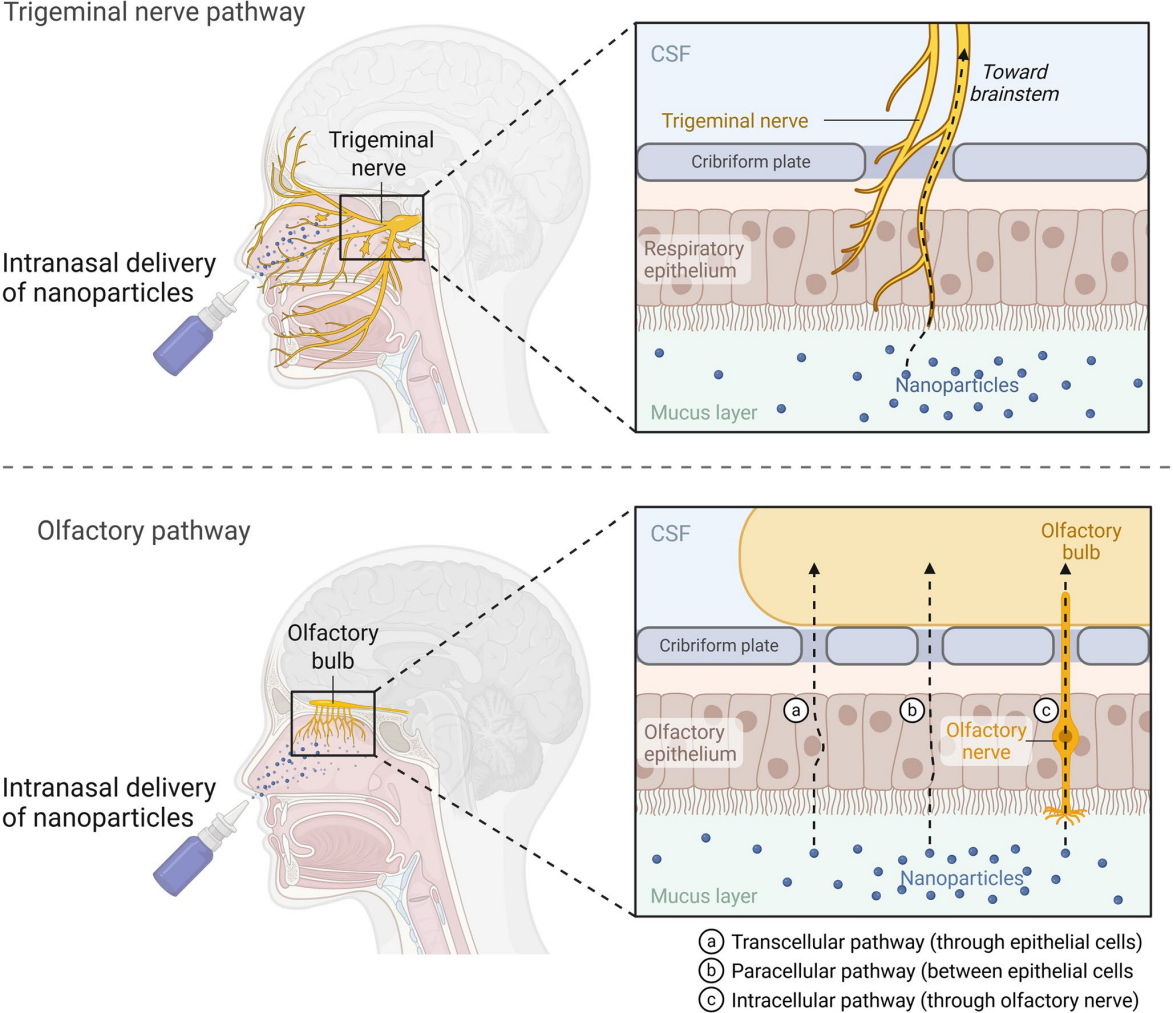
Nose‑to‑brain delivery routes for intranasal nanoparticles. Schematic illustration of the main anatomical routes by which intranasally administered nanoparticles can reach the brain. In the upper panel, particles deposited on the respiratory epithelium cross the mucus layer and are taken up by branches of the trigeminal nerve, which transport them along perineural and/or intracellular pathways toward the brainstem. In the lower panel, particles delivered to the olfactory epithelium access the central nervous system by traversing the epithelial barrier via transcellular (**a**) or paracellular (**b**) routes, or by being internalized and transported along olfactory neurons (**c**), ultimately reaching the cerebrospinal fluid and olfactory bulb. These complementary trigeminal and olfactory pathways enable direct nose‑to‑brain transport while partly bypassing the blood–brain barrier, thereby enhancing the potential of intranasal nanocarriers for targeting deep brain structures such as the hypothalamus

The intranasal route comprises of its own set of barriers that administered nanoparticles should overcome to enter the brain **(**Fig. 2**).** The presence of a thick mucus layer, ciliated cells, and enzymes present the first barrier to penetration which can clear, block and break-down exogenous materials and transport them from the nasal cavity to the gastrointestinal tract, where further degradation could occur [165]. Once these barriers in the vestibular region of the nasal cavity are surpassed, access to the brain occurs through the respiratory and olfactory regions, where the trigeminal and olfactory nerves provide the primary pathways for nose‑to‑brain delivery [165, 166]. Other supporting cells such as the basal cells, goblet cells, ciliated and non-ciliated columnar epithelium also make up the barrier to entry in these regions [165, 166]. It is through these neuronal routes that direct delivery to the pons, cerebrum, olfactory and frontal brain is made possible predominantly either through both transcellular and paracellular transport (Fig. 2). In effect, once the nanoparticles enter the CSF, it can get distributed throughout the brain through bulk flow. To pass through the trigeminal and olfactory nerves (Fig. 2), nanoparticles can be functionalized with appropriate ligands that can enable receptor mediated transcytosis to the brain [166]. For example, one study used lactoferrin‑decorated lipid nanoparticles (LNPs) for Alzheimer’s disease, taking advantage of the fact that lactoferrin receptors are overexpressed in the nasal mucosa and in neurons in this condition [167]. Moreover, the mucolytic agent N-acetyl cysteine was also used to promote nanoparticle penetration and brain bioavailability [168]. With the combined use of targeting and mucolytic agents, enhanced brain accumulation of the nanoparticle was observed as against the use of these agents alone. Other reports have also demonstrated the use of specific ligands such as lectins [169] (that bind to glycan receptors), cell penetrating peptides such as transactivator of transcription (TAT)-derived peptide [170], or other specific ligands (that bind to receptors such as L-fucose [171]) along with mucolytic agents such as hyaluronidase [172] that can enhance nose to brain delivery.

Since the nasal epithelium is usually covered by a thick layer of mucus and contains ciliated cells (Fig. 2) that quickly clear exogenous materials, the use of muco-adhesive materials for the synthesis of nanocarriers has emerged as a rational approach. Under physiological conditions, the mucous layer is slightly acidic with negatively charged mucins, that provide an optimal environment for a polycationic polysaccharide material such as chitosan, to be widely explored for intranasal drug-delivery [173]. Apart from its ability to form positive charges at low pH and its capacity to interact with tight-junction proteins at the nasal epithelium, its biocompatibility and bio-degradability have made it one of the most widely explored material for drug delivery through this route [166]. In addition, due to its versatile physical and chemical properties, chitosan has also been used as a coating material for other nano drug-delivery vehicles to impart muco-adhesive functionality [174–176]. However, it should be noted that strong muco-adhesion could lead to unfavorable retention of nanoparticles in the nasal cavity, and therefore it is essential that the nanoparticles also have the capacity to penetrate through the mucus layer. This is often done through PEGylation of nanoparticle surface in to maintain a balance between muco-adhesion and -penetration [177].

## Nanoparticles for hypothalamic targeting

Due to the proximity of the hypothalamus to the BBB, any exogenous material that surpasses this barrier from the blood stream readily encounters the hypothalamus. On the other hand, as mentioned before, intranasal administration can also provide relatively direct access to the hypothalamus via the olfactory and trigeminal nerve pathways, which enable nose‑to‑brain transport while bypassing the blood-brain barrier [165, 166]. A few reports have evaluated the ability of nanoparticles for brain targeting and its specific effect on the hypothalamus-owing to the important role it plays in maintaining body homeostasis.

Owing to their small size, nanoparticle entry through the BBB without the use of a targeting ligand, is possible in certain cases [178]. This was demonstrated for PAMAM dendrimers, with size < 100 nm and a near-neutral surface charge, that could enter the brain easily post intra-carotid administration [179]. By loading hydrophobic curcumin- a potent anti-oxidant into these dendrimers, their administration to mice produced a shielding effect on the hypothalamus that were exposed to a heat shock condition [179]. This was evidenced through limited neuronal damage as compared to controls, that received the dendrimer nanoparticle alone. In this case, since the protective action of the API is broadly beneficial for the whole hypothalamic region, simple passive diffusion into the BBB is enough to produce the desired effect [179]. However, for specifically targeting the hypothalamus, apart from having the capacity to target and penetrate the BBB, nanoparticles should also include specific ligands on its surface that recognize and bind target cells within the brain tissue. This is important to reduce any non-specific/indiscriminate action of the API within the brain, that can occur due to nanoparticle entry. Thus overall, nanoparticle design for specific targeting within the hypothalamus should take into consideration **(i)** receptor mediated transcytosis for BBB penetration, requiring the need for ligand functionalization **(ii)** cell/site recognition, depending on target region of interest/ diseased condition and **(iii)** cargo to be encapsulated, for example, drug, nucleic acid, peptide etc. Such targeting strategy was utilized for nanoparticles of PEG-PCL co-polymer, that were surface modified with CGN polypeptide (d-CGNHPHLAKYNGT) to penetrate brain capillary endothelial cells [180], and MG polypeptide (CHHSSSAR), that specifically targeted pro-inflammatory microglia [63, 181]. These nanoparticles were demonstrated for the treatment of hypothalamic neuroinflammation [52] by encapsulating and delivering zimlovisertib, an interleukin 1 receptor associated kinase 4 (IRAK4) inhibitor [182]. Similar strategies have also been reported for the treatment of CNS conditions such as Alzheimer’s disease [53] stroke and experimental autoimmune encephalomyelitis in mice [54], where entry and targeting into the brain is necessary.

The dual targeting strategy **(i and ii)** also becomes relevant for intranasal delivery wherein the mucosal barrier needs to be first overcome to penetrate the brain. Specific hypothalamic targeting was demonstrated for nanoparticles administered intranasally in one study wherein a mucolytic agent N-acetyl-L-cysteine was loaded along with a specific receptor binding ligand-neurokinin A, that recognizes neurokinin 3 receptor (N3KR) in neurons of the ARC [183]. By loading these nanoparticles with SB222200, a NK3R antagonist, hypothalamic release of gonadotropin releasing hormone (GnRH) could be regulated [183]. This was demonstrated in a mice model of precocious puberty, where intranasal administration of the designed nanoparticle system led to significant delay in the onset of puberty as compared to controls [183].

In cases where specific receptor mediated hypothalamic targeting is not envisaged, alterations in the structural integrity of the BBB can be exploited for drug delivery. In the case atherosclerosis, where its onset has been linked to the disruption of the BBB [184], untargeted liposomes (encapsulating bromocriptine) when administered intranasally facilitated enhanced BBB penetration and drug accumulation at the hypothalamus, as compared to non-atherosclerotic normal mice [185]. Analysis of brain endothelial cell populations here revealed the presence of hyperproliferative endothelial cells in atherosclerotic mice which also overexpressed organic anion-transporting polypeptide 1a4 (Oatp1a4), a transcellular transporter of amphipathic organic anions, which were hypothesized to cause the increased nanoparticle transport across the BBB [185]. By utilizing this inherent structural anomaly of the BBB in a disease condition, enhanced bromocriptine delivery to the hypothalamus was reported in this study, which ameliorated hyperprolactinemia in a mice model [185].

Another target of interest within the hypothalamus is carnitine palmitoyl-transferase 1 A (CPT1A), an enzyme that regulates the entry of long chain fatty acid into the mitochondria to undergo β-oxidation [186, 187]. By inhibiting CPT1A, an increase in the local concentration of fatty acyl CoA occurs within the hypothalamus, that has been reported to alter its activity leading to increased satiety and reduced food intake in mice [186–188]. This has been taken to advantage by utilizing a fatty acid synthase (FAS) inhibitor, namely C75 (4-methylidene-2-octyl-5-oxooxolane-3-carboxylic acid) [189–192], which is converted to its CoA derivative, C75-CoA in the hypothalamus, where it acts as a potent CPT1 inhibitor and reduces food intake and body weight [193]. Based on that, C75 encapsulated within a polymeric nano-micelle system, and injected intracerebroventricularly, lead to weight loss in mice as compared to free C75 [194]. This provides proof of concept of the importance of CPT1A inhibition in the hypothalamus and its effect on regulating energy metabolism. It would be interesting to carry out further studies to explore the regulation of CPT1A activity through intravenous/intranasal administration of nanoparticles. Alternatively, the targeting of other CPT1 isoforms, such as CPT1C, which is also involved in the hypothalamic regulation of energy balance [187, 195–198], could open new therapeutic avenues for obesity treatment.

## Targeted extracellular vesicles for hypothalamic treatment

Due to the central role of the hypothalamus in regulating energy metabolism, it would be of great advantage to design strategies that will enable the modulation of this regulation, specifically for the treatment of different metabolic disorders [20, 36]. Very few reports have explored this possibility with the use of nanoparticles that can target specific regions within the hypothalamus and have shown encouraging results in metabolic regulation that warrant more research [20, 36].

**Fig. 3:**
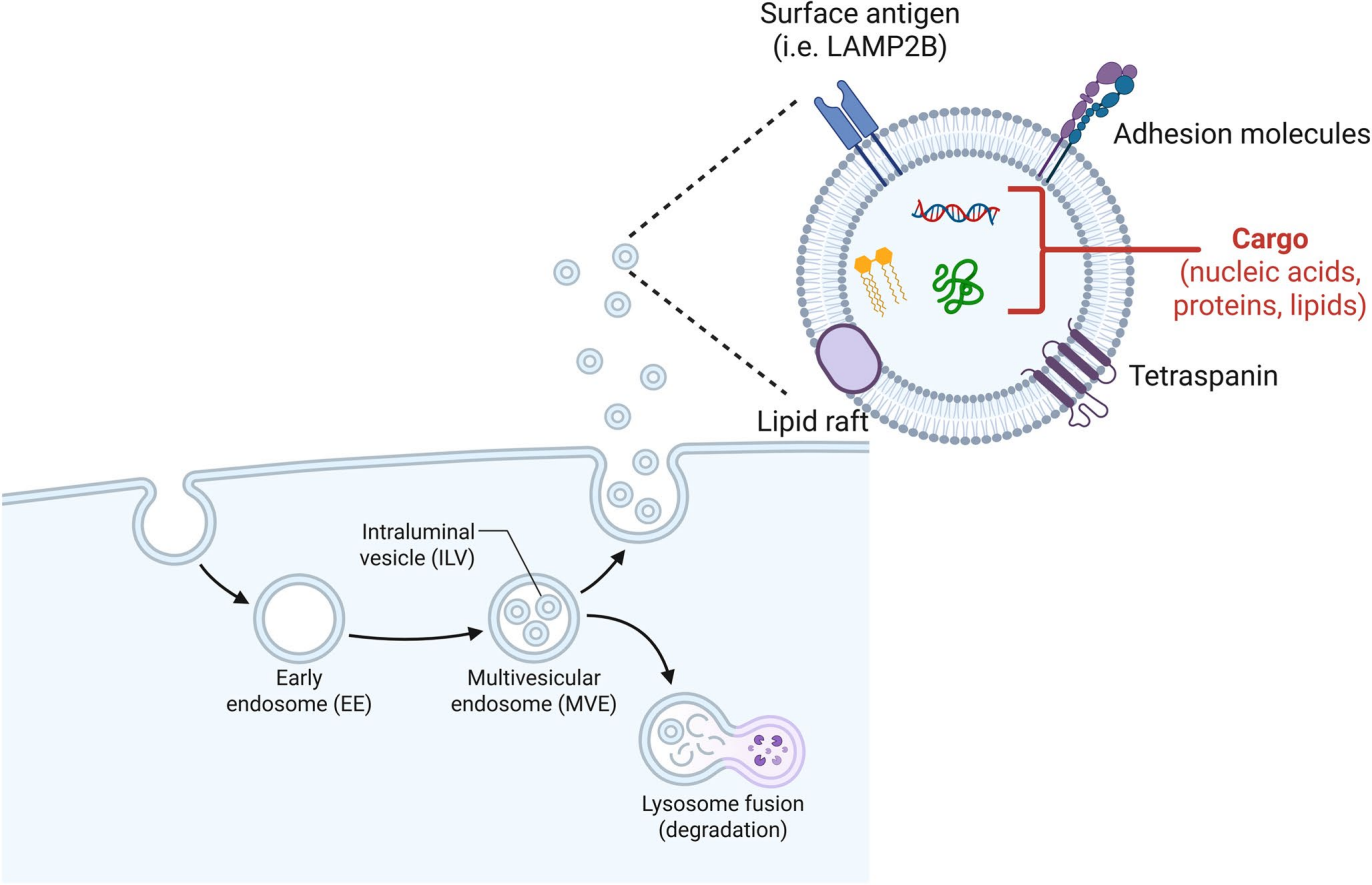
Biogenesis and molecular composition of exosomes/sEVs. Schematic representation of exosome biogenesis through the endosomal pathway. Early endosomes (EE) invaginate their limiting membrane to generate intraluminal vesicles (ILVs) within multivesicular endosomes (MVEs); these MVEs can either fuse with lysosomes for cargo degradation or with the plasma membrane to release ILVs as extracellular vesicles into the extracellular space. The magnified view illustrates the typical molecular composition of an exosome, including surface antigens such as lysosomal-associated membrane protein 2B (LAMP2B), adhesion molecules, lipid rafts and tetraspanins, as well as a complex cargo of nucleic acids, proteins and lipids packaged in the vesicle lumen. Tetraspanins are a family of small four‑pass transmembrane proteins (for example CD9, CD63 and CD81) that organize membrane microdomains, scaffold interaction partners and serve as canonical exosome markers due to their enrichment on the vesicle surface. LAMP2B, a single‑pass lysosome‑associated membrane glycoprotein, can be fused to targeting peptides to display specific antigens on the exosomal surface and thereby direct vesicles to selected cell types or brain regions; a prototypical example is the fusion of LAMP2B to the rabies virus glycoprotein (RVG) peptide, which confers high affinity for nicotinic acetylcholine receptors on brain endothelial cells and neurons and enables systemic delivery of exosomes that preferentially home to the central nervous system

Within this context, exosomes/small extracellular vesicles (sEVs) **(**Fig. 3**)** have gained wide-spread attention as promising biomimetic nano-drug-delivery vehicles owing to their inherent biological function in facilitating intra/inter-cellular communication within the body [20, 36, 199–201]. sEVs are small (30–200 nm in diameter) lipid bilayer-bound vesicles released from living cells into the extracellular environment that lack a functional nuclei and cannot replicate. sEVs originate from the endosomal system, where cargo is first sorted into early endosomes (EE) and then into intraluminal vesicles within multivesicular endosomes (MVE). These MVE can either fuse with lysosomes for degradation or with the plasma membrane, releasing intraluminal vesicles as exosomes into the extracellular space [20, 36, 199–201] **(**Fig. 3**).** They play important roles by transporting bioactive cargo such as nucleic acids, peptides, proteins, chemokines and cytokines and therefore have been linked towards various important functions in the body including maintaining homeostasis [202], regulating disease progression [203, 204], mounting an immunological response [205, 206], etc. Additionally, since sEVs also inherently contain receptor specific ligands on their surface, they have the ability to target and deliver cargo contents to specific cells within the body [207] **(**Fig. 3**)**. Owing to their biomimetic characteristics and small size, they have been investigated for drug delivery. For brain specific delivery, sEVs have been derived from different sources depending upon their targetability and BBB penetrability for the intended application. For the treatment of glioblastoma, for example, neutrophil derived sEVs have been investigated [55], since in an inflammatory microenvironment condition brought about by the presence of a tumor, neutrophils possess the intrinsic ability to penetrate an intact BBB [208]. Upon intravenous injection of doxorubicin loaded sEVs in C6 glioma bearing mice, substantial brain penetration was observed as compared to free dye controls, which indicated that the sEVs actively drove drug entry into the brain [55]. Importantly, when fluorescence imaging of brain sections was undertaken, significant specific sEV localization was observed at the tumor site as compared to the non-tumor sites which highlighted its ability to target the inflammatory micro-environment within the brain [55].

Moreover, since sEVs possess a structure like that of liposomes, reports have highlighted the ability to modify them with additional ligands to enhance brain-specific targeting. This was demonstrated for mesenchymal stem cell derived sEVs for the treatment of ischemic stroke. In order to target the α_v_β_3_ integrins expressed in endothelial cells after ischemia, the mesenchymal cell derived sEVs were further chemically modified to covalently conjugate cyclo(Arg-Gly-Asp-D-Tyr-Lys) peptide [c(RGDyK)] [56]. These reports show how sEVs can be utilized and functionalized if needed for better targeting ability. However, sEV entry into the brain can also be facilitated without the need to target specific cues/diseased condition in the brain. This was demonstrated for blood serum derived sEVs since they inherently contain transferrin receptors on their surface that can bind to free transferrin in blood which itself can bind to transferrin receptors within the BBB epithelium [57]. The versatility of sEVs for drug-delivery to the brain has also been demonstrated using intranasal delivery for the treatment of diseases including ischemia [209], Alzheimer’s disease [210] and Parkinson’s disease [211, 212]. These promising reports warrant their further use for specific targeting to the hypothalamus [44].

Within the hypothalamus, a key region that regulates energy metabolism is the VMH which contains steroidogenic factor 1 (SF1) neurons [13, 16–22]. Downregulation of AMP-activated protein kinase alpha 1 (AMPKα1) subunit activity in these neurons leads to decreased hypothalamic ceramide synthesis and endoplasmic reticulum (ER) stress, increased tone of the sympathetic nervous system (SNS), eliciting brown adipose tissue (BAT)-mediated thermogenesis as well as the browning of white adipose tissue (WAT) [19, 20, 91, 213, 214]. This is a canonical mechanism of hypothalamic regulation of energy balance, integrating central level metabolic and endocrine signals, such as thyroid hormones [87, 88, 215–219], estradiol [93, 220–222], bone morphogenetic protein 8B (BMP8B) [99, 223, 224], leptin [103, 225, 226], and GLP-1 [227], as well as nicotine [228–231]. Of note, specific mediated ablation of AMPKα1 (but not other subunits, such as AMPKγ2) in SF1 neurons protects against HFD-induced obesity and improves metabolism in mice [88, 232, 233]. To inhibit AMPKα1 activity, small extracellular vesicles (sEVs) carrying plasmids encoding a dominant negative AMPKα1 mutant (AMPKα1-DN) for brain targeting was explored [36, 43, 44]. Here, to impart specificity, the plasmid payload also encoded an SF1 promoter sequence which will enable its translation specifically within SF1 neurons [36, 43, 44]. Furthermore, the sEV was also modified to exhibit a rabies virus glycoprotein (RVG) bound to lysosome-associated membrane protein 2b (LAMP2B, a protein highly expressed in sEVs membranes) that would enable nicotinic acetylcholine receptor-mediated trans-cellular entry through the BBB and neuronal targeting [36, 43, 44, 234] **(**Fig. 3**)**. Upon intravenous administration of these sEVs in mice, a significantly high accumulation in the brain was observed for RVG-labelled sEVs as against controls, which underlines the importance of incorporating a targeting agent to cross the BBB [44]. Importantly, in HFD-induced obese mice, the administration of these sEVs leads to substantial weight loss that was found to be induced through thermogenesis of BAT, as well as a tendency for browning of WAT, and was independent of food intake [44]. A similar result was also obtained when these sEVs were used for the treatment of leptin receptor deficiency induced obesity in db/db mice [43]. This highlights the efficacy of this approach wherein specific hypothalamic targeting and regulation is achieved using sEVs. Notably, this strategy has also been explored for the treatment of ischemic stroke in mice. Specifically, mice subjected to transient middle cerebral artery occlusion (tMCAO) when treated with sEVs harboring a dominant negative mutant of the AMPKα2 subunit (AMPKα2-DN) showed not only a reduction of the ischemic area but also cognitive improvement [45]. These findings also demonstrate that the regulation of brain AMPK provides an adequate neuroprotective target for cerebral ischemia, and that the sEV-mediated regulation of this kinase could be a possible clinical strategy against ischemic stroke [45].

Even though sEVs show great versatility for drug delivery, their major drawbacks are related to synthesis and isolation [36, 235, 236], as follows. **(i)** Conventional manufacturing methods for sEVs, such as batch cell culture and ultracentrifugation, are characterized by low yields and substantial variability between production lots, which hampers both fundamental research and practical applications [36, 237–241]. These processes are labor-intensive, time-consuming, and often result in preparations contaminated with other types of extracellular vesicles or cellular debris, compromising the purity and therapeutic efficacy of the final product [36, 237–240]. **(ii)** One of the most pressing issues is the heterogeneity of sEV preparations. Even when sEVs are derived from the same donor cell type, there can be significant variability in size, composition, and biological activity between batches [36, 237–240, 242, 243]. This inconsistency arises from differences in cell culture conditions, passage number, and the inherent variability of biological systems. As a result, ensuring consistent therapeutic potency and safety across production lots remains a critical challenge. **(iii)** The source of sEVs also plays a crucial role in their quality and function. For example, mesenchymal stromal cells (MSCs) are a popular source, but they have limited expansion capacity, necessitating frequent derivation of new cell batches and extensive validation, which is both costly and time-consuming [36, 237–240]. **(iv)** Large-scale production requires expanding anchorage-dependent cells, which is technically demanding and often requires advanced bioreactor systems to maximize yield without altering cell phenotype or sEV functionality [36, 237–240]. Recent advances such as perfusion and batch-refeed culture systems have shown promise in increasing the productivity and quality of sEVs, enabling more consistent production of specific sEV subpopulations over extended periods. These methods can potentially triple the yield of target sEVs and facilitate the maintenance of constant quality attributes, thereby improving reproducibility and scalability [36, 237–240]. However, transitioning from static, planar cultures to dynamic, three-dimensional systems can introduce additional variability, further complicating the maintenance of batch homogeneity. **(v)** Another challenge is quality control and assessment of batch-to-batch consistency. Multiplex bead-based flow cytometry assays have been validated as robust methods for assessing the reproducibility of sEV products, but even with these tools, achieving the required level of consistency for clinical-grade products remains difficult [36, 237–240, 244] Additionally, sEV stability during storage and transport is a concern. Some studies report that sEVs are stable at -80 °C, which is impractical for pharmaceutical logistics, and there is limited data on their shelf-life and in vivo stability. Presently researchers are investigating the use of sEVs derived from abundant sources other than mammalian cell-culture including milk [245, 246], yeast [247–249], plant [250–252] etc. that could be produced in greater amounts at lower costs, and could therefore have greater potential for easier clinical translation.

## Future of metabolic regulation-promising nanomedicine approaches

**Fig. 4:**
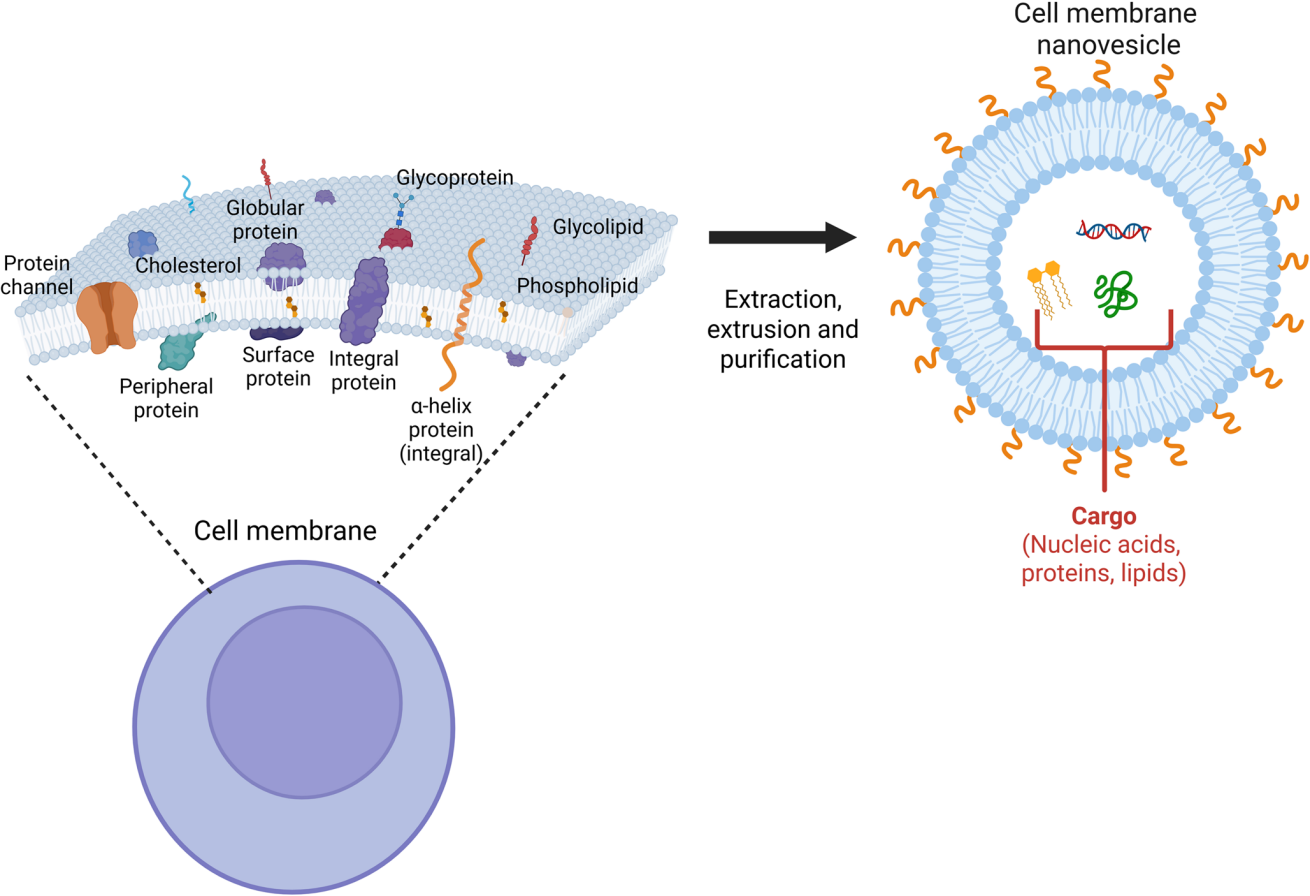
Generation and structure of cell membrane nanovesicles. Schematic representation of cell membrane–derived nanovesicles. Plasma membranes, which contain a complex mixture of phospholipids, cholesterol, glycolipids and diverse membrane proteins (including channels, peripheral and integral proteins, and glycoproteins), are isolated from source cells and subjected to extraction, extrusion and purification steps to form cell membrane nanovesicles. These biomimetic vesicles preserve the native lipid composition and many of the surface proteins of the parental cell, while their lumen can be loaded with therapeutic cargo such as nucleic acids, proteins and lipids. Compared with naturally secreted sEVs, cell membrane nanovesicles can be generated in higher yields by scalable mechanical processes, offering improved batch‑to‑batch reproducibility and more straightforward manufacturing for therapeutic applications

**Fig. 5:**
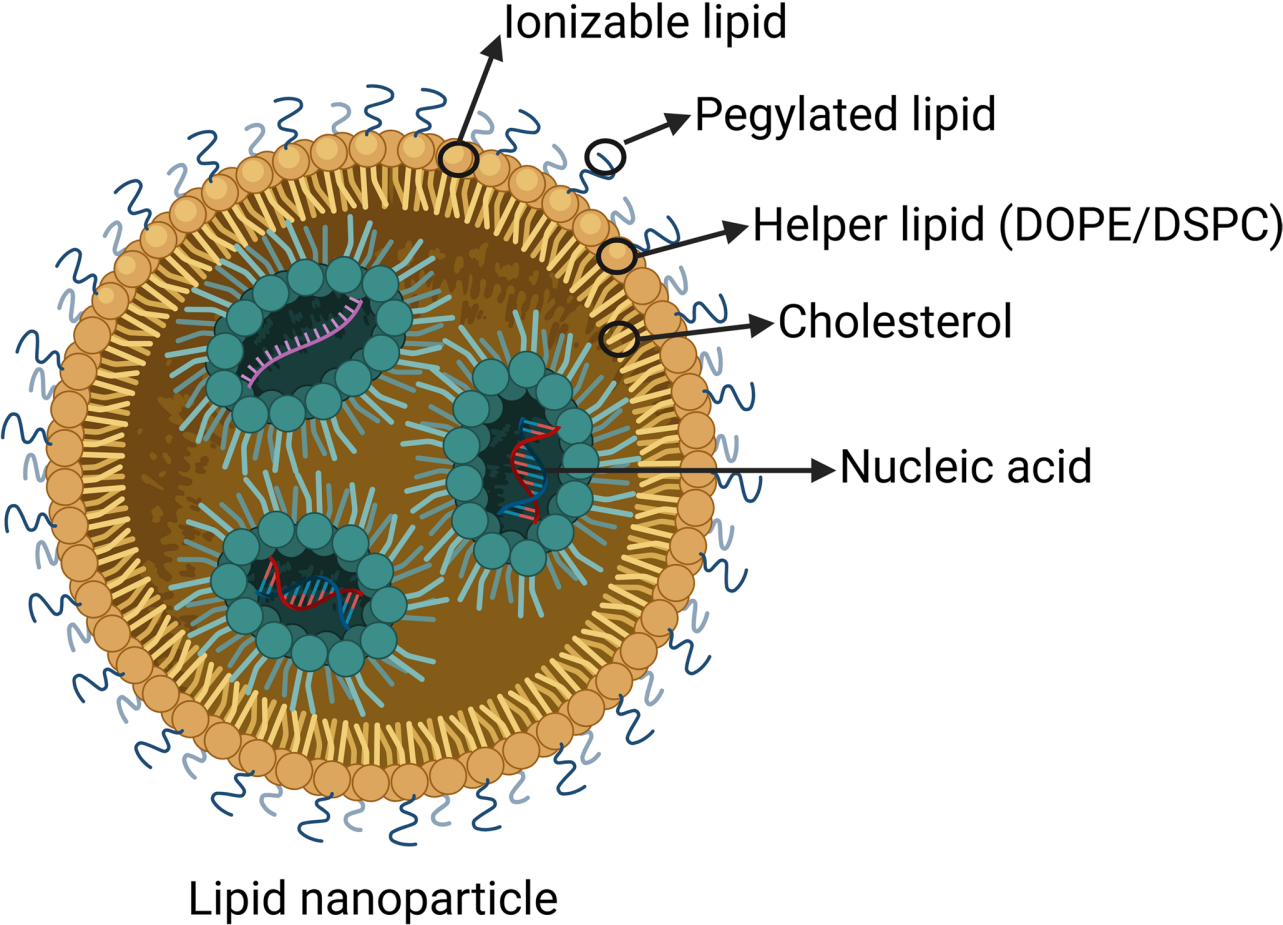
Structural organization and key components of lipid nanoparticles. Schematic representation of a lipid nanoparticle (LNP) formulated for nucleic acid delivery. The particle core contains nucleic acid strands complexed with ionizable lipids, which are positively charged at acidic pH during formulation to efficiently condense and protect the cargo, but become largely neutral at physiological pH to reduce nonspecific interactions and systemic toxicity. The surrounding lipid matrix includes helper lipids such as DOPE or DSPC that stabilize the bilayer and facilitate endosomal escape, cholesterol to modulate membrane fluidity and mechanical rigidity, and an outer shell of PEGylated lipids that improves colloidal stability, prolongs circulation time and reduces opsonization by serum proteins. Together, this modular composition enables high encapsulation efficiency, protection of labile nucleic acids, tuneable pharmacokinetics and scalable manufacturing, making LNPs a particularly attractive platform for mRNA and other nucleic acid therapeutics with potential adaptation for targeted delivery to defined brain and hypothalamic regions

Since nanoparticle-mediated brain targeting has been demonstrated successfully with a few studies exploring specific hypothalamic targeting with the possibility of regulating metabolism [20, 36, 43, 44], it would be great interest to further explore this field for the development of new treatment modalities for metabolic disorders. Within the context of clinical translation from bench to bedside for nanomedicines, it is important to not only consider nanoparticle physicochemical properties when designing new drug delivery systems, but also its biological properties. Here, biocompatibility, toxicity, immunogenicity, and biochemical activity of the developed nano-drug delivery system are vital parameters to consider since these would influence its biodistribution, clearance rate, pharmacokinetics and pharmacodynamic properties once administered inside the body. Among all the nanoparticle systems explored so far, lipid-based nanoparticles have been the most successful in terms of clinical translation due to their excellent biocompatibility, easy chemical tunability, and ability to incorporate various hydrophobic and hydrophilic cargo components. In the present nanomedicine landscape, apart from sEVs, 2 other specific types of lipid-based nanoparticles stand out that show good promise for brain targeting in the future: **(i)** cell-membrane nanovesicles and **(ii)** lipid nanoparticles (LPNs) (Figs. 4 and 5).

### Cell-membrane nanovesicles

With the clear advantages demonstrated by using of sEVs for drug delivery and the challenges that limit their large-scale use and translation, researchers have explored the possibility of utilizing alternate nanovesicle systems that could closely mimic the physicochemical and biological properties of sEVs [42]. Here, cell-membrane nanovesicles, which are synthesized from cell-membranes extracted from a parent cell-source, and re-structured to the nanoscale (~ 100–200 nm), have shown great capacity as sEVs mimics [253–257] **(**Fig. 4**).** Similar to sEVs, these structures exhibit properties close to their parent cell as they display similar protein and carbohydrate ligands that determine their biological activity [258–260]. Importantly, unlike sEVs, the synthesis of cell-membrane nanovesicles do not require energy intensive processes and can be synthesized at a larger scale thereby reducing its production cost and improving clinical translation capability [261]. A typical method for the synthesis of cell-membrane nanovesicles consists of **(i)** cell harvesting, **(ii)** hypo-osmotic treatment to induce cell-swelling (under protease inhibiting conditions), **(iii)** freeze-thaw treatments or homogenization to facilitate cell-disruption, **(iv)** cell-membrane isolation through centrifugation and **(v)** their extrusion through porous membranes to force the formation of nano-sized vesicles, with some variations in these steps reported in literature [262–264] **(**Fig. 4**).** Here, the limiting step to scale-up synthesis of cell-membrane nano-vesicles includes the maintenance of large-scale cell-cultures which could be accomplished through established commercial routes and large-scale cell-extrusion which could be achieved through industrial grade extruders, or through the use of reported modified devices and techniques [265].

To utilize cell-membrane nanovesicles for brain/hypothalamic delivery, the parent cell source from which the cell-membrane is isolated is important as it will play a key role in modulating its activity, pharmacokinetics, and cellular interaction. One strategy is to use cells from the CNS itself. In one example, for developing a treatment strategy for multiple sclerosis, nanovesicles made from extracted myelinated nerve fibers were utilized [266]. Here, the choice of the myelinated nerve fiber membranes was to extract and administer autoantigens in the form of a nanodelivery vehicle to produce an immunological anergy to ameliorate immune dysfunction mediated myelin destruction that occurs in multiple sclerosis [266]. Upon intranasal administration in healthy rats, significant brain penetration was observed for the myelin nanovesicles in 2 h with its presence detected particularly in the olfactory bulbs and cerebellum [266]. A similar targeting strategy was reported for the treatment of ischemic encephalopathy wherein astrocyte cell membrane nanovesicles were used [267]. In these cases, the ability of the cell-membrane nanovesicles to recognize and “home” to its target site is taken to advantage.

Apart from utilizing cells that comprise the brain tissue for the generation of brain targeted nanovesicles, immune cells from the periphery have also been used for targeting the CNS. This was specifically demonstrated for the condition of multiple sclerosis wherein cell membrane nanovesicles from neutrophils were utilized for the treatment of experimental auto-immune encephalomyelitis (EAE) in mice [268]. Upon intravenous administration of these nanovesicles, EAE amelioration was observed which was brought about by improving the phagocytic capacity of microglia leading to better myelin debris clearance [268]. Here, even though a direct evaluation of the penetration capacity of the nanovesicles into the brain was not undertaken, owing to its small size and immune escape capability, its capacity to penetrate deep into the brain should be high. Similar to this, platelet derived cell-membrane nanovesicles have also been demonstrated for the treatment of Alzheimer disease as an example [269]. It is important to note that in such diseased conditions, such as in multiple sclerosis or ischemia, the BBB is considered to be compromised [270, 271], which may enhance the penetration of nanoparticles to the brain. On the other hand, under conditions of metabolic dysfunction the BBB remains intact, and it would be beneficial to include ligands for receptor mediated endocytosis for enhanced brain penetration. Furthermore, the application of external stimuli such as ultra-sound could also be utilized for increasing brain penetration in an otherwise intact BBB [272, 273].

Although endogenous cell-membrane components would be a rational choice for the development of biomimetic nanocarriers, some reports have recently demonstrated the use of exogenous cell-membrane nanovesicles for drug-delivery. Among these, outer membrane vesicles (OMVs) derived from bacteria, similar to sEVs, have shown good potential for the delivery of different types of cargo including nucleic acids, enzymes, proteins, and small molecules [274], and have also shown good promise as possible vaccines owing to the presence of immune activating components such as lipopolysaccharide, lipoteichoic acid, and other bacterial proteins [275]. In one such report, OMVs derived from genetically engineered Escherichia coli BL21 bacteria carrying an under-acylated lipopolysaccharide, which induces lower immune response, was used for drug delivery to the brain [276]. Here, the penetration into the BBB is thought to occur through its interaction with a chaperone glycoprotein 96 expressed on the BBBs endothelial cell surface [277] or through entry after neutrophil uptake [278].

### Lipid nanoparticles

Among all the lipid-based nanoparticle systems till date, the current generation of lipid nanoparticles (LNPs) are the most advanced and clinically successful non-viral nucleic acid delivery system **(**Fig. 5**)**, owing to their success in tackling the COVID-19 pandemic [279]. Their simple synthesis chemistries, easy tunability and scalability and wide nucleic-acid payload capacity [280] have made them one of the most widely researched nanoparticle delivery systems today. Fundamentally, LNPs consist of 4 lipid components: one ionizable cationic lipid, two helper lipids (a phospholipid and cholesterol) and a PEGylated lipid [280, 281] **(**Fig. 2**)**. Here, the ionizable cationic lipid, which contains one or more tertiary amine functional groups, play the most important role in electrostatically interacting with the nucleic acid payload, while the helper lipids and PEGylated lipids primarily provide structural integrity and stability respectively [282]. All 4 lipid components together play an important role in modulating their size, polydispersity, stability, encapsulating efficiency and transfection efficiency among other important parameters [282]. LNPs are synthesized by the simple mixing of the lipid components in ethanol (at fixed molar ratios) with the nucleic acid cargo in an acidic buffer (such as citrate or acetate), which leads to the self-assembly of the lipid components such that the nucleic acid cargo is protected from the outside environment [282]. The low buffer pH leads to the protonation of the ionizable lipid which drives its interaction with the nucleic acid cargo leading to its encapsulation within LNPs. This ionizable cationic lipid also fundamentally enables endosomal escape once they are taken up by cells in vitro or in vivo [282]. Currently researchers are working on developing different types of ionizable cationic lipids that are biodegradable and enable good encapsulation and transfection efficiencies in vivo. One of the critical factors for the success of LNPs worldwide that is often overlooked is their simple synthesis process that can be scaled up easily and quickly with commercially available microfluidic mixers or jet mixers that can enable synthesis from a scale of milliliters to hundreds of liters. All these factors along with the flexibility provided by the nucleic acid cargo for the treatment of different types of diseases have made LNPs one of the most highly efficient and sought out nanomaterial for nucleic acid therapeutics. Apart from nucleic acid cargo, LNPs have also been demonstrated for the delivery of small molecules and protein cargo [283].

Even though LNPs have been highly successful in delivering nucleic acid therapeutics both in vitro and in vivo, this technology still faces the challenge of achieving specific targeting to the desired site of action once administered systemically. At present, intensive efforts are being directed towards the development of organ‑specific and disease‑specific LNP formulations, and several studies have already demonstrated their potential for brain targeting. For example, LNPs conjugated with an antibody against vascular cell adhesion molecule‑1 (VCAM‑1) have been synthesized to target ischemic regions in the brain, taking advantage of the overexpression of VCAM‑1 on endothelial cells under these conditions [48]. In this study, targeted LNPs showed greater brain accumulation than untargeted controls [48], and, importantly, delivery of an IL‑10‑encoding mRNA using these LNPs produced a stronger therapeutic effect than the administration of the anti‑inflammatory drug dexamethasone in targeted liposomes [48, 284]. This superiority was attributed to the “delayed protection” provided by nucleic acid therapeutics, which can sustain protein expression for several days, as opposed to the more transient “early protection” afforded by small‑molecule drugs [48, 284].

As mentioned previously, achieving targeting to specific sites within the brain requires a dual strategy that addresses both the BBB endothelium and the brain region where cargo release is desired. This concept was illustrated in a study on glioblastoma therapy, in which a five‑component LNP formulation was developed; the additional fifth lipid, NT1‑O14B, is an ionizable cationic lipid conjugated to the neurotransmitter tryptamine and was shown to markedly enhance brain delivery [283]. Additionally, a PEGylated lipid functionalized with the Angiopep‑2 peptide, which has high affinity for low‑density lipoprotein receptor‑related protein 1 (LRP1) overexpressed at the BBB and in glioblastoma cells, was used together with a non‑functionalized PEGylated lipid at different ratios [285]. Along with enhanced brain targeting and reduced liver accumulation, maximal brain uptake was achieved when 20–30% of the PEGylated lipid was substituted with Angiopep‑2-functionalized PEGylated lipid, whereas lower (10%) or higher (40%) substitution levels resulted in reduced targeting efficiency [285]. Similar observations were made for RVG29 functionalized LNPs wherein optimum transfection efficiency was observed only at 10% substitution of PEGylated lipid with peptide [286]. Overall, the addition of the Angiopep-2 targeting agent led to a brain accumulation of 2.23% injected dose which was 1.35 times higher than its non-targeted control [286]. A similar approach was utilized in another report, where an ionizable lipid OS4T was synthesized that was chemically bound to the ligand of serotonin receptor (5-hydroxytryptamine type 3), that had the ability to cross the BBB. The synthesized LNPs showed optimum transfection efficiency only when a particular molar ratio of 40:40:60:0.75 (Ionizable lipid/DOPE/Chol/DMG-PEG2k) was used. Furthermore, when different cell penetrating peptides including vascular endothelial cadherin derived peptide (pVEC), RVG, and transactivator of transcription peptide (Tat) were conjugated onto the LNPs, only the Tat CPP showed maximum brain penetration with nucleic acid delivery observed in neurons, astrocytes, microglia, and brain capillary endothelial cells [287]. This study as well as others [287, 288] show how careful optimization of LNP synthesis and ligand selection play a key role in achieving organ specific targeting.

One exciting upcoming application of LNPs is its ability to deliver genome editing machinery in vivo [289–292]. LNPs were demonstrated for the efficient association of Cre-recombinase enzyme as well as the Cas9-sgRNA ribonucleoprotein machinery [134]. Here, while the Cas9-sgRNA was inherently anionic (which enabled its electrostatic interaction with the ionizable cationic lipid), the Cre-recombinase cargo was fused with a modified negatively supercharged green fluorescent protein (GFP), that enabled its association to LNPs. By utilizing an ionizable cationic lipid with different amine head groups and hydrophobic chain lengths, and by modulating the anionic charge of the GFP protein to alter encapsulation efficiency, an optimized formulation was determined which showed maximum protein delivery and gene recombination efficiency in in vitro conditions [134]. Upon intra-thecal administration of the optimized formulation in specific regions of the brain in Rosa^26tdTomato^ mouse, strong TdTomato signal could be observed at the injected sites 6 days post administration highlighting its ability to successfully undertake gene editing in vivo [134]. Importantly, it was shown that gene editing was possible in extremely small regions of the brain, which opens the possibilities of treating various neurological diseases [134]. This, and other studies utilizing LNPs showcase the tremendous opportunities that could be taken to advantage through gene editing [289–292] and nucleic-acid delivery [48, 285, 286] and could perform very well in targeting the hypothalamus to enable regulation of energy homeostasis and treatment of various metabolic disorders.

## Conclusions

The hypothalamus sits at the core of whole-body energy homeostasis, integrating peripheral hormonal, nutrient, and neural cues to orchestrate feeding behavior, energy expenditure, and neuroendocrine responses [13, 16–21]. Its dysfunction contributes to obesity, insulin resistance, type 2 diabetes, and other metabolic disorders [13, 16–21], yet direct therapeutic modulation of hypothalamic circuits remains technically challenging [20, 36]. In parallel, nanomedicine has matured to the point where clinically approved nanocarriers and increasingly sophisticated targeting strategies can be rationally engineered for brain delivery [20, 23–25, 29, 36]. Bringing these two fields together opens a largely unexplored but highly promising avenue for metabolic disease intervention.

Current nanoparticle platforms already enable efficient crossing of the BBB through receptor-mediated transcytosis, disease-associated BBB remodeling, or nose-to-brain routes, and can be further refined to reach specific hypothalamic nuclei or cell types [20, 23–25, 29, 36]. By combining tailored materials, surface ligands, and controlled release properties, nanocarriers can deliver small molecules, peptides, or nucleic acids to modulate key hypothalamic pathways such as AMPK [20, 36, 43–45], CPT1A activity [194], inflammatory cascades, or neuropeptide networks. Adapting brain‑penetrant cell-membrane nanovesicles and LNPs to selectively modulate defined hypothalamic targets such as AMPK and CPT1A, or specific neuronal populations including POMC and agouti-related peptide (AgRP) neurons and pro‑inflammatory microglia, represents a key unmet goal. Achieving this level of precision would enable neurometabolic interventions that go beyond current systemic therapies and could transform the treatment of obesity and related metabolic disorders.

However, despite this potential, several hurdles must be overcome before hypothalamus-focused nanotherapies can reach the clinic. These include **(i)** improving selectivity for defined neuronal and glial populations, **(ii)** minimizing off-target accumulation and long-term toxicity, **(iii)** standardizing large-scale and reproducible manufacturing, and **(iv)** developing robust imaging and biomarker strategies to monitor target engagement in humans. Integration of human genetics, advanced neuroimaging, and circuit-level physiology with rational nanocarrier design will be essential to identify the most relevant targets and delivery routes for specific metabolic indications. Overall, the convergence of hypothalamic biology and nanodelivery technologies will offer a unique opportunity to move beyond systemic pharmacology towards precision neurometabolic interventions, and merits concerted multidisciplinary efforts in the coming years.

## Data Availability

No datasets were generated or analysed during the current study.

## References

[1] Zhang H, Zhou XD, Shapiro MD, Lip GYH, Tilg H, Valenti L, Somers VK, Byrne CD, Targher G, Yang W, et al. Global burden of metabolic diseases, 1990–2021. Metabolism. 2024;160:155999. 39151887 10.1016/j.metabol.2024.155999

[2] Chew NWS, Ng CH, Tan DJH, Kong G, Lin C, Chin YH, Lim WH, Huang DQ, Quek J, Fu CE, et al. The global burden of metabolic disease: data from 2000 to 2019. Cell Metab. 2023;35(3):414–28. e413. 36889281 10.1016/j.cmet.2023.02.003

[3] Holm SK, Johansen VBI, Clemmensen C. LEAP2 as a therapeutic target in obesity and cardiometabolic disorders. Rev Endocr Metab Disord 2025. 10.1007/s11154-025-10007-4 41284160

[4] Garus-Pakowska A. Metabolic Diseases-A challenge for public health in the 21st century. Int J Environ Res Public Health. 2023;20(18):6789. 37754648 10.3390/ijerph20186789 PMC10530887

[5] Collaboration NCDRF. Worldwide trends in underweight and obesity from 1990 to 2022: a pooled analysis of 3663 population-representative studies with 222 million children, adolescents, and adults. Lancet. 2024;403(10431):1027–50. 38432237 10.1016/S0140-6736(23)02750-2 PMC7615769

[6] Collaboration NCDRF. Trends in adult body-mass index in 200 countries from 1975 to 2014: a pooled analysis of 1698 population-based measurement studies with 19.2 million participants. Lancet. 2016;387(10026):1377–96. 27115820 10.1016/S0140-6736(16)30054-X PMC7615134

[7] Loos RJF, Yeo GSH. The genetics of obesity: from discovery to biology. Nat Rev Genet. 2022;23(2):120–33. 34556834 10.1038/s41576-021-00414-z PMC8459824

[8] Kusminski CM, Perez-Tilve D, Muller TD, DiMarchi RD, Tschop MH, Scherer PE. Transforming obesity: the advancement of multi-receptor drugs. Cell. 2024;187(15):3829–53. 39059360 10.1016/j.cell.2024.06.003 PMC11286204

[9] Friedman JM. On the causes of obesity and its treatment: the end of the beginning. Cell Metab. 2025;37(3):570–7. 40043689 10.1016/j.cmet.2025.01.026

[10] Kokkorakis M, Chakhtoura M, Rhayem C, Al Rifai J, Ghezzawi M, Valenzuela-Vallejo L, Mantzoros CS. Emerging pharmacotherapies for obesity: A systematic review. Pharmacol Rev. 2025;77(1):100002. 39952695 10.1124/pharmrev.123.001045

[11] Muller TD. Advances in incretin-based drug discovery in 2025. Nat Rev Endocrinol. 2025;22(2):68–9. 10.1038/s41574-025-01219-4 41388157

[12] Dragano NRV, Ferno J, Dieguez C, Lopez M, Milbank E. Recent updates on obesity treatments: available drugs and future directions. Neuroscience. 2020;437:215–39. 32360593 10.1016/j.neuroscience.2020.04.034

[13] Argente J, Farooqi IS, Chowen JA, Kuhnen P, Lopez M, Morselli E, Gan HW, Spoudeas HA, Wabitsch M, Tena-Sempere M. Hypothalamic obesity: from basic mechanisms to clinical perspectives. Lancet Diabetes Endocrinol. 2025;13(1):57–68. 39547253 10.1016/S2213-8587(24)00283-3

[14] Clemmensen C, Finan B, Muller TD, DiMarchi RD, Tschop MH, Hofmann SM. Emerging hormonal-based combination pharmacotherapies for the treatment of metabolic diseases. Nat Rev Endocrinol. 2019;15(2):90–104. 30446744 10.1038/s41574-018-0118-x

[15] Muller TD, Bluher M, Tschop MH, DiMarchi RD. Anti-obesity drug discovery: advances and challenges. Nat Rev Drug Discov. 2022;21(3):201–23. 34815532 10.1038/s41573-021-00337-8 PMC8609996

[16] Bruning JC, Fenselau H. Integrative neurocircuits that control metabolism and food intake. Science. 2023;381(6665):eabl7398. 37769095 10.1126/science.abl7398

[17] Lee TH, Nicolas JC, Quarta C. Molecular and functional mapping of the neuroendocrine hypothalamus: a new era begins. J Endocrinol Invest. 2024;47(11):2627–48. 38878127 10.1007/s40618-024-02411-5

[18] Schneeberger M, Gomis R, Claret M. Hypothalamic and brainstem neuronal circuits controlling homeostatic energy balance. J Endocrinol. 2014;220(2):T25–46. 24222039 10.1530/JOE-13-0398

[19] Lopez M, Nogueiras R, Tena-Sempere M, Dieguez C. Hypothalamic AMPK: a canonical regulator of whole-body energy balance. Nat Rev Endocrinol. 2016;12(7):421–32. 27199291 10.1038/nrendo.2016.67

[20] Lopez M. Hypothalamic AMPK as a possible target for energy balance-related diseases. Trends Pharmacol Sci. 2022;43(7):546–56. 35570061 10.1016/j.tips.2022.04.007

[21] Le Thuc O, Garcia-Caceres C. Obesity-induced inflammation: connecting the periphery to the brain. Nat Metab. 2024;6(7):1237–52. 38997442 10.1038/s42255-024-01079-8

[22] Lopez M, Tena-Sempere M, Dieguez C. Cross-talk between orexins (hypocretins) and the neuroendocrine axes (hypothalamic-pituitary axes). Front Neuroendocrinol. 2010;31(2):113–27. 19654017 10.1016/j.yfrne.2009.07.001

[23] Terstappen GC, Meyer AH, Bell RD, Zhang W. Strategies for delivering therapeutics across the blood-brain barrier. Nat Rev Drug Discov. 2021;20(5):362–83. 33649582 10.1038/s41573-021-00139-y

[24] Wu D, Chen Q, Chen X, Han F, Chen Z, Wang Y. The blood-brain barrier: structure, regulation, and drug delivery. Signal Transduct Target Ther. 2023;8(1):217. 37231000 10.1038/s41392-023-01481-w PMC10212980

[25] Saraiva C, Praca C, Ferreira R, Santos T, Ferreira L, Bernardino L. Nanoparticle-mediated brain drug delivery: overcoming blood-brain barrier to treat neurodegenerative diseases. J Control Release. 2016;235:34–47. 27208862 10.1016/j.jconrel.2016.05.044

[26] Nampoothiri S, Nogueiras R, Schwaninger M, Prevot V. Glial cells as integrators of peripheral and central signals in the regulation of energy homeostasis. Nat Metab. 2022;4(7):813–25. 35879459 10.1038/s42255-022-00610-z PMC7613794

[27] Chen B, de Launoit E, Meseguer D, Garcia Caceres C, Eichmann A, Renier N, Schneeberger M. The interactions between energy homeostasis and neurovascular plasticity. Nat Rev Endocrinol. 2024;20(12):749–59. 39054359 10.1038/s41574-024-01021-8

[28] Garcia-Caceres C, Balland E, Prevot V, Luquet S, Woods SC, Koch M, Horvath TL, Yi CX, Chowen JA, Verkhratsky A, et al. Role of astrocytes, microglia, and tanycytes in brain control of systemic metabolism. Nat Neurosci. 2019;22(1):7–14. 30531847 10.1038/s41593-018-0286-y

[29] Korbelin J, Arrulo A, Schwaninger M. Gene therapy targeting the blood-brain barrier. Vitam Horm. 2024;126:191–217. 39029973 10.1016/bs.vh.2024.03.001

[30] Chen B, Furtado J, Schneeberger M. Neurovascular plasticity as a modulator of hypothalamic function. Rev Endocr Metab Disord. 2025. 10.1007/s11154-025-09982-5 40542999

[31] Steuernagel L, Lam BYH, Klemm P, Dowsett GKC, Bauder CA, Tadross JA, Hitschfeld TS, Del Rio Martin A, Chen W, de Solis AJ, et al. HypoMap-a unified single-cell gene expression atlas of the murine hypothalamus. Nat Metab. 2022;4(10):1402–19. 36266547 10.1038/s42255-022-00657-y PMC9584816

[32] Tadross JA, Steuernagel L, Dowsett GKC, Kentistou KA, Lundh S, Porniece M, Klemm P, Rainbow K, Hvid H, Kania K, et al. A comprehensive spatio-cellular map of the human hypothalamus. Nature. 2025;639(8055):708–16. 39910307 10.1038/s41586-024-08504-8 PMC11922758

[33] Mukherjee S, Skrede S, Ferno J. The hypothalamus as a therapeutic target: towards novel approaches for managing antipsychotic-induced weight gain. Rev Endocr Metab Disord. 2025. 10.1007/s11154-025-10009-2 PMC13246901 41432886

[34] Campbell JN, Macosko EZ, Fenselau H, Pers TH, Lyubetskaya A, Tenen D, Goldman M, Verstegen AM, Resch JM, McCarroll SA, et al. A molecular census of arcuate hypothalamus and median eminence cell types. Nat Neurosci. 2017;20(3):484–96. 28166221 10.1038/nn.4495 PMC5323293

[35] Nance E. Brain-Penetrating nanoparticles for analysis of the brain microenvironment. Methods Mol Biol. 2017;1570:91–104. 28238131 10.1007/978-1-4939-6840-4_6

[36] Mukherjee S, Dieguez C, Ferno J, Lopez M. Obesity wars: hypothalamic sEVs a new hope. Trends Mol Med. 2023;29(8):622–34. 37210227 10.1016/j.molmed.2023.04.006

[37] Mitchell MJ, Billingsley MM, Haley RM, Wechsler ME, Peppas NA, Langer R. Engineering precision nanoparticles for drug delivery. Nat Rev Drug Discov. 2021;20(2):101–24. 33277608 10.1038/s41573-020-0090-8 PMC7717100

[38] Mendes BB, Conniot J, Avital A, Yao D, Jiang X, Zhou X, Sharf-Pauker N, Xiao Y, Adir O, Liang H, et al. Nanodelivery of nucleic acids. Nat Rev Methods Primers. 2022;2:24. 35480987 10.1038/s43586-022-00104-y PMC9038125

[39] Hou X, Zaks T, Langer R, Dong Y. Lipid nanoparticles for mRNA delivery. Nat Rev Mater. 2021;6(12):1078–94. 34394960 10.1038/s41578-021-00358-0 PMC8353930

[40] Beach MA, Nayanathara U, Gao Y, Zhang C, Xiong Y, Wang Y, Such GK. Polymeric nanoparticles for drug delivery. Chem Rev. 2024;124(9):5505–616. 38626459 10.1021/acs.chemrev.3c00705 PMC11086401

[41] Sun L, Liu H, Ye Y, Lei Y, Islam R, Tan S, Tong R, Miao YB, Cai L. Smart nanoparticles for cancer therapy. Signal Transduct Target Ther. 2023;8(1):418. 37919282 10.1038/s41392-023-01642-x PMC10622502

[42] Chen Y, Douanne N, Wu T, Kaur I, Tsering T, Erzingatzian A, Nadeau A, Juncker D, Nerguizian V, Burnier JV. Leveraging nature’s nanocarriers: translating insights from extracellular vesicles to biomimetic synthetic vesicles for biomedical applications. Sci Adv. 2025;11(9):eads5249. 40009680 10.1126/sciadv.ads5249 PMC11864201

[43] Milbank E, Dragano N, Vidal-Gomez X, Rivas-Limeres V, Garrido-Gil P, Wertheimer M, Recoquillon S, Pata MP, Labandeira-Garcia JL, Dieguez C, et al. Small extracellular vesicle targeting of hypothalamic AMPKalpha1 promotes weight loss in leptin receptor deficient mice. Metabolism. 2023;139:155350. 36423694 10.1016/j.metabol.2022.155350

[44] Milbank E, Dragano NRV, Gonzalez-Garcia I, Garcia MR, Rivas-Limeres V, Perdomo L, Hilairet G, Ruiz-Pino F, Mallegol P, Morgan DA, et al. Small extracellular vesicle-mediated targeting of hypothalamic AMPKalpha1 corrects obesity through BAT activation. Nat Metab. 2021;3(10):1415–31. 34675439 10.1038/s42255-021-00467-8

[45] Ouro A, Rodriguez-Diaz A, Lopez-Gonzalez T, Romaus-Sanjurjo D, Estevez-Salguero A, Iglesias-Rey R, Rodriguez-Arrizabalaga M, Fernandez-Sanmartin P, Castro-Mosquera M, Debasa-Mouce M, et al. Neuroprotective effect of small extracellular vesicle-mediated targeting of AMPKalpha2 in cerebral ischemia. Metabolism. 2025;167:156160. 39961478 10.1016/j.metabol.2025.156160 PMC12005051

[46] Lochhead JJ, Thorne RG. Intranasal delivery of biologics to the central nervous system. Adv Drug Deliv Rev. 2012;64(7):614–28. 22119441 10.1016/j.addr.2011.11.002

[47] Chen Y, Zhang C, Huang Y, Ma Y, Song Q, Chen H, Jiang G, Gao X. Intranasal drug delivery: the interaction between nanoparticles and the nose-to-brain pathway. Adv Drug Deliv Rev. 2024;207:115196. 38336090 10.1016/j.addr.2024.115196

[48] Nong J, Glassman PM, Shuvaev VV, Reyes-Esteves S, Descamps HC, Kiseleva RY, Papp TE, Alameh MG, Tam YK, Mui BL, et al. Targeting lipid nanoparticles to the blood-brain barrier to ameliorate acute ischemic stroke. Mol Ther. 2024;32(5):1344–58. 38454606 10.1016/j.ymthe.2024.03.004 PMC11081939

[49] Lu G, Wang X, Li F, Wang S, Zhao J, Wang J, Liu J, Lyu C, Ye P, Tan H, et al. Engineered biomimetic nanoparticles achieve targeted delivery and efficient metabolism-based synergistic therapy against glioblastoma. Nat Commun. 2022;13(1):4214. 35864093 10.1038/s41467-022-31799-y PMC9304377

[50] Hou K, Zhao J, Wang H, Li B, Li K, Shi X, Wan K, Ai J, Lv J, Wang D, et al. Chiral gold nanoparticles enantioselectively rescue memory deficits in a mouse model of alzheimer’s disease. Nat Commun. 2020;11(1):4790. 32963242 10.1038/s41467-020-18525-2 PMC7509831

[51] van Vliet EF, Knol MJ, Schiffelers RM, Caiazzo M, Fens M. Levodopa-loaded nanoparticles for the treatment of parkinson’s disease. J Control Release. 2023;360:212–24. 37343725 10.1016/j.jconrel.2023.06.026

[52] Goo YT, Grigoriev V, Korzun T, Sharma KS, Singh P, Taratula OR, Marks DL, Taratula O. Blood-Brain Barrier-Penetrating nanocarriers enable Microglial-Specific drug delivery in hypothalamic neuroinflammation. Adv Healthc Mater. 2025;14(13):e2500521. 40181631 10.1002/adhm.202500521 PMC12084131

[53] Zheng X, Pang X, Yang P, Wan X, Wei Y, Guo Q, Zhang Q, Jiang X. A hybrid SiRNA delivery complex for enhanced brain penetration and precise amyloid plaque targeting in alzheimer’s disease mice. Acta Biomater. 2017;49:388–401. 27845275 10.1016/j.actbio.2016.11.029

[54] Yin N, Zhao Y, Liu C, Yang Y, Wang ZH, Yu W, Zhang K, Zhang Z, Liu J, Zhang Y, et al. Engineered nanoerythrocytes alleviate central nervous system inflammation by regulating the polarization of inflammatory microglia. Adv Mater. 2022;34(27):e2201322. 35483045 10.1002/adma.202201322

[55] Wang J, Tang W, Yang M, Yin Y, Li H, Hu F, Tang L, Ma X, Zhang Y, Wang Y. Inflammatory tumor microenvironment responsive neutrophil exosomes-based drug delivery system for targeted glioma therapy. Biomaterials. 2021;273:120784. 33848731 10.1016/j.biomaterials.2021.120784

[56] Tian T, Zhang HX, He CP, Fan S, Zhu YL, Qi C, Huang NP, Xiao ZD, Lu ZH, Tannous BA, et al. Surface functionalized exosomes as targeted drug delivery vehicles for cerebral ischemia therapy. Biomaterials. 2018;150:137–49. 29040874 10.1016/j.biomaterials.2017.10.012

[57] Cui J, Wang X, Li J, Zhu A, Du Y, Zeng W, Guo Y, Di L, Wang R. Immune exosomes loading Self-Assembled nanomicelles traverse the Blood-Brain barrier for Chemo-immunotherapy against glioblastoma. ACS Nano. 2023;17(2):1464–84. 10.1021/acsnano.2c10219 36626296

[58] Liang Y, Iqbal Z, Lu J, Wang J, Zhang H, Chen X, Duan L, Xia J. Cell-derived nanovesicle-mediated drug delivery to the brain: principles and strategies for vesicle engineering. Mol Ther. 2023;31(5):1207–24. 36245129 10.1016/j.ymthe.2022.10.008 PMC10188644

[59] Jimenez-Puyer M, Sobrino V, Colledge WH, Jones S, Tena-Sempere M. Hypothalamic control of puberty: from neuronal circuits to mechanisms for its metabolic regulation. Rev Endocr Metab Disord. 2025. 10.1007/s11154-025-10001-w PMC13246832 41108362

[60] Gonzalez-Garcia I, Xu Y. Hypothalamic actions of estrogens in the regulation of energy and glucose homeostasis. Rev Endocr Metab Disord. 2025. 10.1007/s11154-025-09994-1 PMC13246899 41231413

[61] Littleton SH, Tadross JA, Yeo GSH. From identity to function: unveiling the cellular complexity of hypothalamic feeding circuits. Rev Endocr Metab Disord. 2025. 10.1007/s11154-025-10004-7 PMC7618707 41148441

[62] Mittag J, Kolms B. Hypothalamic control of heart rate and body temperature by thyroid hormones. Rev Endocr Metab Disord. 2025. 10.1007/s11154-025-09966-5 40244498

[63] Zagmutt S, Rodriguez-Garcia M, Bolanos-Hurtado M, Reguera AC, Casals N, Rodriguez-Rodriguez R. Redefining the timeline: a three-phase framework of hypothalamic microinflammation in metabolic disease. Rev Endocr Metab Disord. 2025. 10.1007/s11154-025-09992-3 PMC13246836 41065953

[64] Farooqi IS, Keogh JM, Yeo GS, Lank EJ, Cheetham T, O’Rahilly S. Clinical spectrum of obesity and mutations in the melanocortin 4 receptor gene. N Engl J Med. 2003;348(12):1085–95. 12646665 10.1056/NEJMoa022050

[65] Goodarzi MO. Genetics of obesity: what genetic association studies have taught Us about the biology of obesity and its complications. Lancet Diabetes Endocrinol. 2018;6(3):223–36. 28919064 10.1016/S2213-8587(17)30200-0

[66] Jouque V, Miralpeix C, Lopez-Gambero AJ, Nicolas JC, Quarta C, Cota D. Beyond satiety: unraveling the complex roles of POMC neurons in behavior and metabolism. Rev Endocr Metab Disord. 2025. 10.1007/s11154-025-09993-2 PMC13246545 40971005

[67] Quarta C, Claret M, Zeltser LM, Williams KW, Yeo GSH, Tschop MH, Diano S, Bruning JC, Cota D. POMC neuronal heterogeneity in energy balance and beyond: an integrated view. Nat Metab. 2021;3(3):299–308. 33633406 10.1038/s42255-021-00345-3 PMC8085907

[68] Claret M, Haddad-Tovolli R. Sexual dimorphism in the development and function of the melanocortin system. Rev Endocr Metab Disord. 2025. 10.1007/s11154-025-09983-4 40555875

[69] Faour M, Mesto N, Martin C, Luquet S. Emerging role of AgRP neurons as integrators of metabolic, sensory and environmental cues in the control of energy homeostasis. Rev Endocr Metab Disord. 2025. 10.1007/s11154-025-09990-5 PMC13246522 40762661

[70] Clement K, Vaisse C, Lahlou N, Cabrol S, Pelloux V, Cassuto D, Gourmelen M, Dina C, Chambaz J, Lacorte JM, et al. A mutation in the human leptin receptor gene causes obesity and pituitary dysfunction. Nature. 1998;392(6674):398–401. 9537324 10.1038/32911

[71] Hung CC, Luan J, Sims M, Keogh JM, Hall C, Wareham NJ, O’Rahilly S, Farooqi IS. Studies of the SIM1 gene in relation to human obesity and obesity-related traits. Int J Obes (Lond). 2007;31(3):429–34. 16924270 10.1038/sj.ijo.0803443

[72] Michaud JL, Boucher F, Melnyk A, Gauthier F, Goshu E, Levy E, Mitchell GA, Himms-Hagen J, Fan CM. Sim1 haploinsufficiency causes hyperphagia, obesity and reduction of the paraventricular nucleus of the hypothalamus. Hum Mol Genet. 2001;10(14):1465–73. 11448938 10.1093/hmg/10.14.1465

[73] Stijnen P, Ramos-Molina B, O’Rahilly S, Creemers JW. PCSK1 mutations and human endocrinopathies: from obesity to Gastrointestinal disorders. Endocr Rev. 2016;37(4):347–71. 27187081 10.1210/er.2015-1117

[74] Speakman JR. The ‘Fat mass and obesity related’ (FTO) gene: mechanisms of impact on obesity and energy balance. Curr Obes Rep. 2015;4(1):73–91. 26627093 10.1007/s13679-015-0143-1

[75] Gerken T, Girard CA, Tung YC, Webby CJ, Saudek V, Hewitson KS, Yeo GS, McDonough MA, Cunliffe S, McNeill LA, et al. The obesity-associated FTO gene encodes a 2-oxoglutarate-dependent nucleic acid demethylase. Science. 2007;318(5855):1469–72. 17991826 10.1126/science.1151710 PMC2668859

[76] Tung YCL, Yeo GSH, O’Rahilly S, Coll AP. Obesity and FTO: changing focus at a complex locus. Cell Metab. 2014;20(5):710–8. 25448700 10.1016/j.cmet.2014.09.010

[77] Posey KA, Clegg DJ, Printz RL, Byun J, Morton GJ, Vivekanandan-Giri A, Pennathur S, Baskin DG, Heinecke JW, Woods SC, et al. Hypothalamic Proinflammatory lipid accumulation, inflammation, and insulin resistance in rats fed a high-fat diet. Am J Physiol Endocrinol Metab. 2009;296(5):E1003–1012. 19116375 10.1152/ajpendo.90377.2008 PMC2681305

[78] Thaler JP, Yi CX, Schur EA, Guyenet SJ, Hwang BH, Dietrich MO, Zhao X, Sarruf DA, Izgur V, Maravilla KR, et al. Obesity is associated with hypothalamic injury in rodents and humans. J Clin Invest. 2012;122(1):153–62. 22201683 10.1172/JCI59660 PMC3248304

[79] Valdearcos M, Douglass JD, Robblee MM, Dorfman MD, Stifler DR, Bennett ML, Gerritse I, Fasnacht R, Barres BA, Thaler JP et al. Microglial inflammatory signaling orchestrates the hypothalamic immune response to dietary excess and mediates obesity susceptibility. Cell Metab. 2017;26(1):185–197.e183. 10.1016/j.cmet.2017.05.015 PMC5569901 28683286

[80] Sewaybricker LE, Huang A, Chandrasekaran S, Melhorn SJ, Schur EA. The significance of hypothalamic inflammation and gliosis for the pathogenesis of obesity in humans. Endocr Rev. 2023;44(2):281–96. 36251886 10.1210/endrev/bnac023 PMC10216879

[81] Dragano NRV, Milbank E, Haddad-Tovolli R, Garrido-Gil P, Novoa E, Fondevilla MF, Capelli V, Zanesco AM, Solon C, Morari J, et al. Hypothalamic free fatty acid receptor-1 regulates whole-body energy balance. Mol Metab. 2024;79:101840. 38036170 10.1016/j.molmet.2023.101840 PMC10784317

[82] Zhang X, Zhang G, Zhang H, Karin M, Bai H, Cai D. Hypothalamic IKKbeta/NF-kappaB and ER stress link overnutrition to energy imbalance and obesity. Cell. 2008;135(1):61–73. 18854155 10.1016/j.cell.2008.07.043 PMC2586330

[83] Konner AC, Bruning JC. Selective insulin and leptin resistance in metabolic disorders. Cell Metab. 2012;16(2):144–52. 22883229 10.1016/j.cmet.2012.07.004

[84] Gupta S, Knight AG, Gupta S, Keller JN, Bruce-Keller AJ. Saturated long-chain fatty acids activate inflammatory signaling in astrocytes. J Neurochem. 2012;120(6):1060–71. 22248073 10.1111/j.1471-4159.2012.07660.x PMC3296820

[85] Douglass JD, Dorfman MD, Fasnacht R, Shaffer LD, Thaler JP. Astrocyte IKKbeta/NF-kappaB signaling is required for diet-induced obesity and hypothalamic inflammation. Mol Metab. 2017;6(4):366–73. 28377875 10.1016/j.molmet.2017.01.010 PMC5369266

[86] De Souza CT, Araujo EP, Bordin S, Ashimine R, Zollner RL, Boschero AC, Saad MJ, Velloso LA. Consumption of a fat-rich diet activates a Proinflammatory response and induces insulin resistance in the hypothalamus. Endocrinology. 2005;146(10):4192–9. 16002529 10.1210/en.2004-1520

[87] Fernandez-Gonzalez I, Freire-Agulleiro O, Ferreira V, Silveira-Loureiro M, Rial-Pensado E, Garrido-Gil P, Martinez G, Rada P, Labandeira-Garcia JL, Mittag J, et al. JNK1 in SF1 neurons regulates the central action of thyroid hormones on hepatic lipid metabolism. Mol Metab. 2025;98:102170. 40419019 10.1016/j.molmet.2025.102170 PMC12173623

[88] Martinez-Sanchez N, Seoane-Collazo P, Contreras C, Varela L, Villarroya J, Rial-Pensado E, Buque X, Aurrekoetxea I, Delgado TC, Vazquez-Martinez R, et al. Hypothalamic AMPK-ER Stress-JNK1 axis mediates the central actions of thyroid hormones on energy balance. Cell Metab. 2017;26(1):212–29. e212. 28683288 10.1016/j.cmet.2017.06.014 PMC5501726

[89] Won JC, Jang PG, Namkoong C, Koh EH, Kim SK, Park JY, Lee KU, Kim MS. Central administration of an Endoplasmic reticulum stress inducer inhibits the anorexigenic effects of leptin and insulin. Obes (Silver Spring). 2009;17(10):1861–5. 10.1038/oby.2009.194 19543218

[90] Mayer CM, Belsham DD. Palmitate attenuates insulin signaling and induces Endoplasmic reticulum stress and apoptosis in hypothalamic neurons: rescue of resistance and apoptosis through adenosine 5’ monophosphate-activated protein kinase activation. Endocrinology. 2010;151(2):576–85. 19952270 10.1210/en.2009-1122

[91] Contreras C, Gonzalez-Garcia I, Martinez-Sanchez N, Seoane-Collazo P, Jacas J, Morgan DA, Serra D, Gallego R, Gonzalez F, Casals N, et al. Central ceramide-induced hypothalamic lipotoxicity and ER stress regulate energy balance. Cell Rep. 2014;9(1):366–77. 25284795 10.1016/j.celrep.2014.08.057 PMC5157160

[92] Contreras C, Gonzalez-Garcia I, Seoane-Collazo P, Martinez-Sanchez N, Linares-Pose L, Rial-Pensado E, Ferno J, Tena-Sempere M, Casals N, Dieguez C, et al. Reduction of hypothalamic Endoplasmic reticulum stress activates Browning of white fat and ameliorates obesity. Diabetes. 2017;66(1):87–99. 27634226 10.2337/db15-1547

[93] Gonzalez-Garcia I, Contreras C, Estevez-Salguero A, Ruiz-Pino F, Colsh B, Pensado I, Linares-Pose L, Rial-Pensado E, Martinez de Morentin PB, Ferno J, et al. Estradiol regulates energy balance by ameliorating hypothalamic Ceramide-Induced ER stress. Cell Rep. 2018;25(2):413–23. e415. 30304681 10.1016/j.celrep.2018.09.038 PMC6198289

[94] Linares-Pose L, Rial-Pensado E, Estevez-Salguero A, Milbank E, Gonzalez-Garcia I, Rodriguez C, Seoane-Collazo P, Martinez-Sanchez N, Nogueiras R, Prieto D, et al. Genetic targeting of GRP78 in the VMH improves obesity independently of food intake. Genes (Basel). 2018;9(7):357. 30018241 10.3390/genes9070357 PMC6070933

[95] Feng Z, Fang C, Ma Y, Chang J. Obesity-induced blood-brain barrier dysfunction: phenotypes and mechanisms. J Neuroinflammation. 2024;21(1):110. 38678254 10.1186/s12974-024-03104-9 PMC11056074

[96] Davanzo GG, Castro G, Monteiro LB, Castelucci BG, Jaccomo VH, da Silva FC, Marques AM, Francelin C, de Campos BB, de Aguiar CF, et al. Obesity increases blood-brain barrier permeability and aggravates the mouse model of multiple sclerosis. Mult Scler Relat Disord. 2023;72:104605. 36907120 10.1016/j.msard.2023.104605

[97] Schur EA, Melhorn SJ, Oh SK, Lacy JM, Berkseth KE, Guyenet SJ, Sonnen JA, Tyagi V, Rosalynn M, De Leon B, et al. Radiologic evidence that hypothalamic gliosis is associated with obesity and insulin resistance in humans. Obes (Silver Spring). 2015;23(11):2142–8. 10.1002/oby.21248 PMC4634110 26530930

[98] Guo DF, Zhao Y, Laule C, Rahmouni K. BBSome: an essential component of hypothalamic regulation of energy homeostasis. Rev Endocr Metab Disord. 2025. 10.1007/s11154-025-09979-0 PMC13246823 40560450

[99] Rial-Pensado E, Freire-Agulleiro O, Rios M, Guo DF, Contreras C, Seoane-Collazo P, Tovar S, Nogueiras R, Dieguez C, Rahmouni K, et al. Obesity induces resistance to central action of BMP8B through a mechanism involving the BBSome. Mol Metab. 2022;59:101465. 35218946 10.1016/j.molmet.2022.101465 PMC8933534

[100] Rouabhi M, Guo DF, Morgan DA, Zhu Z, Lopez M, Zingman L, Grobe JL, Rahmouni K. BBSome ablation in SF1 neurons causes obesity without comorbidities. Mol Metab. 2021;48:101211. 33722691 10.1016/j.molmet.2021.101211 PMC8065214

[101] Guo DF, Lin Z, Wu Y, Searby C, Thedens DR, Richerson GB, Usachev YM, Grobe JL, Sheffield VC, Rahmouni K. The BBSome in POMC and AgRP neurons is necessary for body weight regulation and sorting of metabolic receptors. Diabetes. 2019;68(8):1591–603. 31127052 10.2337/db18-1088 PMC6692817

[102] Guo DF, Cui H, Zhang Q, Morgan DA, Thedens DR, Nishimura D, Grobe JL, Sheffield VC, Rahmouni K. The BBSome controls energy homeostasis by mediating the transport of the leptin receptor to the plasma membrane. PLoS Genet. 2016;12(2):e1005890. 26926121 10.1371/journal.pgen.1005890 PMC4771807

[103] Cui H, Lopez M, Rahmouni K. The cellular and molecular bases of leptin and Ghrelin resistance in obesity. Nat Rev Endocrinol. 2017;13(6):338–51. 28232667 10.1038/nrendo.2016.222 PMC8904083

[104] Bombassaro B, Araujo EP, Velloso LA. The hypothalamus as the central regulator of energy balance and its impact on current and future obesity treatments. Arch Endocrinol Metab. 2024;68(Spec Issue):e240082. 39876968 10.20945/2359-4292-2024-0082 PMC11771753

[105] Tran LT, Park S, Kim SK, Lee JS, Kim KW, Kwon O. Hypothalamic control of energy expenditure and thermogenesis. Exp Mol Med. 2022;54(4):358–69. 35301430 10.1038/s12276-022-00741-z PMC9076616

[106] Lopaschuk GD, Ussher JR, Jaswal JS. Targeting intermediary metabolism in the hypothalamus as a mechanism to regulate appetite. Pharmacol Rev. 2010;62(2):237–64. 20392806 10.1124/pr.109.002428

[107] Chen J, Cai M, Zhan C. Neuronal regulation of feeding and energy metabolism: A focus on the hypothalamus and brainstem. Neurosci Bull. 2025;41(4):665–75. 39704987 10.1007/s12264-024-01335-7 PMC11978587

[108] Waterson MJ, Horvath TL. Neuronal regulation of energy homeostasis: beyond the hypothalamus and feeding. Cell Metab. 2015;22(6):962–70. 26603190 10.1016/j.cmet.2015.09.026

[109] Patra JK, Das G, Fraceto LF, Campos EVR, Rodriguez-Torres MDP, Acosta-Torres LS, Diaz-Torres LA, Grillo R, Swamy MK, Sharma S, et al. Nano based drug delivery systems: recent developments and future prospects. J Nanobiotechnol. 2018;16(1):71. 10.1186/s12951-018-0392-8 PMC6145203 30231877

[110] Zhang RX, Li J, Zhang T, Amini MA, He C, Lu B, Ahmed T, Lip H, Rauth AM, Wu XY. Importance of integrating nanotechnology with Pharmacology and physiology for innovative drug delivery and therapy - an illustration with firsthand examples. Acta Pharmacol Sin. 2018;39(5):825–44. 29698389 10.1038/aps.2018.33 PMC5943915

[111] Lin N, Tan K, Wei Y, Xie S, Liu J, Ma X. The progress and perspective of nanomedicine in modulating the tumor immune microenvironment. Acta Pharm Sinica B. 2026;16(1):137–68. 10.1016/j.apsb.2025.10.002 PMC12827901 41584347

[112] Zalba S, Ten Hagen TLM, Burgui C, Garrido MJ. Stealth nanoparticles in oncology: facing the PEG dilemma. J Control Release. 2022;351:22–36. 36087801 10.1016/j.jconrel.2022.09.002

[113] Gravan P, Pena-Martin J, de Andres JL, Pedrosa M, Villegas-Montoya M, Galisteo-Gonzalez F, Marchal JA, Sanchez-Moreno P. Exploring the impact of nanoparticle stealth coatings in cancer models: from pegylation to cell Membrane-Coating nanotechnology. ACS Appl Mater Interfaces. 2024;16(2):2058–74. 38159050 10.1021/acsami.3c13948 PMC10797597

[114] Gomerdinger VF, Nabar N, Hammond PT. Advancing engineering design strategies for targeted cancer nanomedicine. Nat Rev Cancer. 2025;25(9):657–83. 40751005 10.1038/s41568-025-00847-2

[115] Veiga N, Diesendruck Y, Peer D. Targeted nanomedicine: lessons learned and future directions. J Control Release. 2023;355:446–57. 36773958 10.1016/j.jconrel.2023.02.010

[116] Choi YH, Han HK. Nanomedicines: current status and future perspectives in aspect of drug delivery and pharmacokinetics. J Pharm Investig. 2018;48(1):43–60. 30546919 10.1007/s40005-017-0370-4 PMC6244736

[117] Van Herck S, De Geest BG. Nanomedicine-mediated alteration of the Pharmacokinetic profile of small molecule cancer immunotherapeutics. Acta Pharmacol Sin. 2020;41(7):881–94. 32451411 10.1038/s41401-020-0425-3 PMC7471422

[118] Dai XJ, Li WJ, Xie DD, Liu BX, Gong L, Han HH. Stimuli-Responsive nano drug delivery systems for the treatment of neurological diseases. Small. 2025;21(9):e2410030. 39840482 10.1002/smll.202410030

[119] Blum AP, Kammeyer JK, Rush AM, Callmann CE, Hahn ME, Gianneschi NC. Stimuli-responsive nanomaterials for biomedical applications. J Am Chem Soc. 2015;137(6):2140–54. 25474531 10.1021/ja510147n PMC4353031

[120] Hauser AK, Wydra RJ, Stocke NA, Anderson KW, Hilt JZ. Magnetic nanoparticles and nanocomposites for remote controlled therapies. J Control Release. 2015;219:76–94. 26407670 10.1016/j.jconrel.2015.09.039 PMC4669063

[121] Linsley CS, Wu BM. Recent advances in light-responsive on-demand drug-delivery systems. Ther Deliv. 2017;8(2):89–107. 28088880 10.4155/tde-2016-0060 PMC5561969

[122] Sia CS, Tey BT, Low LE. Light-Responsive nanoassemblies: advancing biomedical innovation. Adv Funct Mater. 2024;34(23):2314278.

[123] Sana B, Finne-Wistrand A, Pappalardo D. Recent development in near infrared light-responsive polymeric materials for smart drug-delivery systems. Mater Today Chem. 2022;25:100963.

[124] Entzian K, Aigner A. Drug delivery by Ultrasound-Responsive nanocarriers for cancer treatment. Pharmaceutics. 2021;13(8):1135. 34452096 10.3390/pharmaceutics13081135 PMC8397943

[125] Chen Z, Sang L, Qixi Z, Li X, Liu Y, Bai Z. Ultrasound-responsive nanoparticles for imaging and therapy of brain tumors. Mater Today Bio. 2025;32:101661. 40206140 10.1016/j.mtbio.2025.101661 PMC11979416

[126] Gao W, Chan JM, Farokhzad OC. pH-Responsive nanoparticles for drug delivery. Mol Pharm. 2010;7(6):1913–20. 20836539 10.1021/mp100253e PMC3379544

[127] Mu Y, Gong L, Peng T, Yao J, Lin Z. Advances in pH-responsive drug delivery systems. OpenNano. 2021;5:100031.

[128] Nanotechnology-based medicinal. products for human use [https://www.ema.europa.eu/en/documents/report/nanotechnology-based-medicinal-products-human-use-eu-horizon-scanning-report_en.pdf

[129] Li SD, Huang L. Pharmacokinetics and biodistribution of nanoparticles. Mol Pharm. 2008;5(4):496–504. 18611037 10.1021/mp800049w

[130] Wang Y, Wang J, Zhu D, Wang Y, Qing G, Zhang Y, Liu X, Liang XJ. Effect of physicochemical properties on in vivo fate of nanoparticle-based cancer immunotherapies. Acta Pharm Sin B. 2021;11(4):886–902. 33996405 10.1016/j.apsb.2021.03.007 PMC8105773

[131] Cai X, Jin M, Yao L, He B, Ahmed S, Safdar W, Ahmad I, Cheng DB, Lei Z, Sun T. Physicochemical properties, pharmacokinetics, toxicology and application of nanocarriers. J Mater Chem B. 2023;11(4):716–33. 36594785 10.1039/d2tb02001g

[132] Fowler MJ, Cotter JD, Knight BE, Sevick-Muraca EM, Sandberg DI, Sirianni RW. Intrathecal drug delivery in the era of nanomedicine. Adv Drug Deliv Rev. 2020;165–166:77–95. 32142739 10.1016/j.addr.2020.02.006 PMC8182643

[133] Khang M, Lee JH, Lee T, Suh HW, Lee S, Cavaliere A, Rushing A, Geraldo LH, Belitzky E, Rossano S, et al. Intrathecal delivery of nanoparticle PARP inhibitor to the cerebrospinal fluid for the treatment of metastatic Medulloblastoma. Sci Transl Med. 2023;15(720):eadi1617. 37910601 10.1126/scitranslmed.adi1617 PMC11078331

[134] Wang M, Zuris JA, Meng F, Rees H, Sun S, Deng P, Han Y, Gao X, Pouli D, Wu Q, et al. Efficient delivery of genome-editing proteins using bioreducible lipid nanoparticles. Proc Natl Acad Sci U S A. 2016;113(11):2868–73. 26929348 10.1073/pnas.1520244113 PMC4801296

[135] Sun F, Shen H, Yang Q, Yuan Z, Chen Y, Guo W, Wang Y, Yang L, Bai Z, Liu Q, et al. Dual behavior regulation: Tether-Free Deep-Brain stimulation by photothermal and upconversion hybrid nanoparticles. Adv Mater. 2023;35(21):e2210018. 36864009 10.1002/adma.202210018

[136] Kumar M, Kulkarni P, Liu S, Chemuturi N, Shah DK. Nanoparticle biodistribution coefficients: A quantitative approach for Understanding the tissue distribution of nanoparticles. Adv Drug Deliv Rev. 2023;194:114708. 36682420 10.1016/j.addr.2023.114708

[137] Wehn AC, Krestel E, Harapan BN, Klymchenko A, Plesnila N, Khalin I. To see or not to see: in vivo nanocarrier detection methods in the brain and their challenges. J Control Release. 2024;371:216–36. 38810705 10.1016/j.jconrel.2024.05.044

[138] Yousfan A, Al Rahwanji MJ, Hanano A, Al-Obaidi H. A comprehensive study on nanoparticle drug delivery to the brain: application of machine learning techniques. Mol Pharm. 2024;21(1):333–45. 38060692 10.1021/acs.molpharmaceut.3c00880 PMC10762658

[139] Gonzalez-Carter D, Liu X, Tockary TA, Dirisala A, Toh K, Anraku Y, Kataoka K. Targeting nanoparticles to the brain by exploiting the blood-brain barrier impermeability to selectively label the brain endothelium. Proc Natl Acad Sci U S A. 2020;117(32):19141–50. 32703811 10.1073/pnas.2002016117 PMC7431083

[140] van Rooy I, Cakir-Tascioglu S, Hennink WE, Storm G, Schiffelers RM, Mastrobattista E. In vivo methods to study uptake of nanoparticles into the brain. Pharm Res. 2011;28(3):456–71. 20924653 10.1007/s11095-010-0291-7 PMC3044085

[141] Lo EH, Singhal AB, Torchilin VP, Abbott NJ. Drug delivery to damaged brain. Brain Res Brain Res Rev. 2001;38(1–2):140–8. 11750930 10.1016/s0165-0173(01)00083-2

[142] Martinez-Martinez S, Iglesias C, Dieguez C, Rasika S, Schwaninger M, Prevot V, Nogueiras R. The role of tanycytes in the regulation of energy balance. Rev Endocr Metab Disord. 2025. 10.1007/s11154-025-10010-9 41460475

[143] Langlet F, Levin BE, Luquet S, Mazzone M, Messina A, Dunn-Meynell AA, Balland E, Lacombe A, Mazur D, Carmeliet P, et al. Tanycytic VEGF-A boosts blood-hypothalamus barrier plasticity and access of metabolic signals to the arcuate nucleus in response to fasting. Cell Metab. 2013;17(4):607–17. 23562080 10.1016/j.cmet.2013.03.004 PMC3695242

[144] Morita S, Miyata S. Accessibility of low-molecular-mass molecules to the median eminence and arcuate hypothalamic nucleus of adult mouse. Cell Biochem Funct. 2013;31(8):668–77. 23348371 10.1002/cbf.2953

[145] Le Thuc O, Lechner FM, Garcia-Caceres C. Hypothalamic astrocytes: connecting brain and periphery in metabolic control. Rev Endocr Metab Disord. 2025. 10.1007/s11154-025-09995-0 PMC13246531 41082168

[146] Morita-Takemura S, Wanaka A. Blood-to-brain communication in the hypothalamus for energy intake regulation. Neurochem Int. 2019;128:135–42. 31002894 10.1016/j.neuint.2019.04.007

[147] Johnsen KB, Burkhart A, Thomsen LB, Andresen TL, Moos T. Targeting the transferrin receptor for brain drug delivery. Prog Neurobiol. 2019;181:101665. 31376426 10.1016/j.pneurobio.2019.101665

[148] Betzer O, Shilo M, Opochinsky R, Barnoy E, Motiei M, Okun E, Yadid G, Popovtzer R. The effect of nanoparticle size on the ability to cross the blood-brain barrier: an in vivo study. Nanomed (Lond). 2017;12(13):1533–46. 10.2217/nnm-2017-0022 28621578

[149] Boado RJ, Lu JZ, Hui EK, Pardridge WM. Insulin receptor antibody-sulfamidase fusion protein penetrates the primate blood-brain barrier and reduces glycosoaminoglycans in Sanfilippo type A cells. Mol Pharm. 2014;11(8):2928–34. 24949884 10.1021/mp500258p PMC4137762

[150] Ohtsuki S. Insulin receptor at the blood-brain barrier: transport and signaling. Vitam Horm. 2024;126:113–24. 39029970 10.1016/bs.vh.2024.05.001

[151] Laabs M, Mulac D, Langer K. Targeting the blood-brain barrier with lipoprotein-mimicking nanoparticles loaded with Flurbiprofenaxetil and coated with Apolipoprotein E(3). Eur J Pharm Sci. 2025;214:107272. 40972159 10.1016/j.ejps.2025.107272

[152] Di L, Maiseyeu A. Low-density lipoprotein nanomedicines: mechanisms of targeting, biology, and theranostic potential. Drug Deliv. 2021;28(1):408–21. 33594923 10.1080/10717544.2021.1886199 PMC7894439

[153] Pawar S, Koneru T, McCord E, Tatiparti K, Sau S, Iyer AK. LDL receptors and their role in targeted therapy for glioma: a review. Drug Discov Today. 2021;26(5):1212–25. 33609780 10.1016/j.drudis.2021.02.008

[154] Mhaske A, Shukla S, Ahirwar K, Singh KK, Shukla R. Receptor-Assisted nanotherapeutics for overcoming the Blood-Brain barrier. Mol Neurobiol. 2024;61(11):8702–38. 38558360 10.1007/s12035-024-04015-9 PMC11496374

[155] Bao Q, Hu P, Xu Y, Cheng T, Wei C, Pan L, Shi J. Simultaneous Blood-Brain barrier crossing and protection for stroke treatment based on Edaravone-Loaded ceria nanoparticles. ACS Nano. 2018;12(7):6794–805. 29932327 10.1021/acsnano.8b01994

[156] Wiley DT, Webster P, Gale A, Davis ME. Transcytosis and brain uptake of transferrin-containing nanoparticles by tuning avidity to transferrin receptor. Proc Natl Acad Sci U S A. 2013;110(21):8662–7. 23650374 10.1073/pnas.1307152110 PMC3666717

[157] Kuo YC, Ko HF. Targeting delivery of saquinavir to the brain using 83 – 14 monoclonal antibody-grafted solid lipid nanoparticles. Biomaterials. 2013;34(20):4818–30. 23545288 10.1016/j.biomaterials.2013.03.013

[158] Ruan H, Chen X, Xie C, Li B, Ying M, Liu Y, Zhang M, Zhang X, Zhan C, Lu W, et al. Stapled RGD peptide enables Glioma-Targeted drug delivery by overcoming multiple barriers. ACS Appl Mater Interfaces. 2017;9(21):17745–56. 28497694 10.1021/acsami.7b03682

[159] Guo X, Deng G, Liu J, Zou P, Du F, Liu F, Chen AT, Hu R, Li M, Zhang S, et al. Thrombin-Responsive, Brain-Targeting nanoparticles for improved stroke therapy. ACS Nano. 2018;12(8):8723–32. 30107729 10.1021/acsnano.8b04787

[160] Wu H, Peng B, Mohammed FS, Gao X, Qin Z, Sheth KN, Zhou J, Jiang Z. Brain Targeting, antioxidant polymeric nanoparticles for stroke drug delivery and therapy. Small. 2022;18(22):e2107126. 35306743 10.1002/smll.202107126 PMC9167795

[161] Lin T, Zhao P, Jiang Y, Tang Y, Jin H, Pan Z, He H, Yang VC, Huang Y. Blood-Brain-Barrier-Penetrating albumin nanoparticles for biomimetic drug delivery via albumin-Binding protein pathways for antiglioma therapy. ACS Nano. 2016;10(11):9999–10012. 27934069 10.1021/acsnano.6b04268

[162] Ji Q, Zhu H, Qin Y, Zhang R, Wang L, Zhang E, Zhou X, Meng R. GP60 and SPARC as albumin receptors: key targeted sites for the delivery of antitumor drugs. Front Pharmacol. 2024;15:1329636. 38323081 10.3389/fphar.2024.1329636 PMC10844528

[163] Hanson LR, Frey WH 2. Intranasal delivery bypasses the blood-brain barrier to target therapeutic agents to the central nervous system and treat neurodegenerative disease. BMC Neurosci. 2008;9(Suppl 3):S5. 19091002 10.1186/1471-2202-9-S3-S5 PMC2604883

[164] Drath I, Richter F, Feja M. Nose-to-brain drug delivery: from bench to bedside. Transl Neurodegener. 2025;14(1):23. 40390100 10.1186/s40035-025-00481-w PMC12090632

[165] Agrawal M, Saraf S, Saraf S, Antimisiaris SG, Chougule MB, Shoyele SA, Alexander A. Nose-to-brain drug delivery: an update on clinical challenges and progress towards approval of anti-Alzheimer drugs. J Control Release. 2018;281:139–77. 29772289 10.1016/j.jconrel.2018.05.011

[166] Bolon M, Fieux M, Monge C, Richard S. Polymeric nanoparticles for efficient nose-to-brain delivery. Nanoscale. 2025;17(31):17947–79. 40693481 10.1039/d5nr00870k

[167] Xu Y, Ye X, Du Y, Yang W, Tong F, Li W, Huang Q, Chen Y, Li H, Gao H, et al. Nose-to-brain delivery of targeted lipid nanoparticles as two-pronged beta-amyloid nanoscavenger for alzheimer’s disease therapy. Acta Pharm Sin B. 2025;15(6):2884–99. 40654363 10.1016/j.apsb.2025.02.035 PMC12254852

[168] Banerjee S, McCormack S. Acetylcysteine for patients requiring mucous secretion clearance: A review of clinical effectiveness and safety. Ottawa (ON); 2019. 31503431

[169] Chen J, Zhang C, Liu Q, Shao X, Feng C, Shen Y, Zhang Q, Jiang X. Solanum tuberosum lectin-conjugated PLGA nanoparticles for nose-to-brain delivery: in vivo and in vitro evaluations. J Drug Target. 2012;20(2):174–84. 21992548 10.3109/1061186X.2011.622396

[170] Kanazawa T, Taki H, Tanaka K, Takashima Y, Okada H. Cell-penetrating peptide-modified block copolymer micelles promote direct brain delivery via intranasal administration. Pharm Res. 2011;28(9):2130–9. 21499835 10.1007/s11095-011-0440-7

[171] Yang X, Yang W, Xia X, Lei T, Yang Z, Jia W, Zhou Y, Cheng G, Gao H. Intranasal delivery of BACE1 SiRNA and Rapamycin by dual targets modified nanoparticles for alzheimer’s disease therapy. Small. 2022;18(30):e2203182. 35771092 10.1002/smll.202203182

[172] Jia Y, Xu L, Leng S, Sun Y, Huang X, Wang Y, Ren H, Li G, Bai Y, Zhang Z, et al. Nose-to-Brain delivery of circular RNA SCMH1-Loaded lipid nanoparticles for ischemic stroke therapy. Adv Mater. 2025;37(19):e2500598. 40143778 10.1002/adma.202500598

[173] Qureshi M, Aqil M, Imam SS, Ahad A, Sultana Y. Formulation and evaluation of neuroactive drug loaded Chitosan nanoparticle for nose to brain delivery: In-vitro characterization and In-vivo behavior study. Curr Drug Deliv. 2019;16(2):123–35. 30317997 10.2174/1567201815666181011121750

[174] de Barros CT, Alves TFR, Crescencio KMM, Asami J, Hausen MA, Duek EAR, Chaud MV. Chitosan-Coated liposomes for intranasal delivery of ghrelin: enhancing bioavailability to the central nervous system. Pharmaceutics. 2025;17(11):1493. 41304829 10.3390/pharmaceutics17111493 PMC12655848

[175] Sharma S, Gauba P, Tyagi A, Dang S. Chitosan-modified polymeric nanoparticles for the nose-to-brain drug delivery of paroxetine: an in vitro and in vivo evaluation. Nanoscale. 2025;17(3):1687–702. 39641172 10.1039/d4nr04250f

[176] Prabakaran A, Rakshit D, Patel I, Susanna KJ, Mishra A, Radhakrishnanand P, Sarma P, Alexander A. Enhanced cognitive function in mice through intranasal delivery of sinapic acid via chitosan-coated solid lipid nanoparticles. Int J Pharm. 2025;675:125565. 40187700 10.1016/j.ijpharm.2025.125565

[177] Gao X, Xiong Y, Chen H, Gao X, Dai J, Zhang Y, Zou W, Gao Y, Jiang Z, Han B. Mucus adhesion vs. mucus penetration? Screening nanomaterials for nasal inhalation by MD simulation. J Control Release. 2023;353:366–79. 36462640 10.1016/j.jconrel.2022.11.051

[178] Asimakidou E, Tan JKS, Zeng J, Lo CH. Blood-brain barrier-targeting nanoparticles: biomaterial properties and biomedical applications in translational neuroscience. Pharmaceuticals (Basel). 2024;17(5):612. 38794182 10.3390/ph17050612 PMC11123901

[179] Guo F, Wu Y, Liu J. Curcumin nanoparticles in heat stroke management. J Nanobiotechnol. 2024;22(1):559. 10.1186/s12951-024-02771-3 PMC11396141 39267043

[180] Guo Q, Xu S, Yang P, Wang P, Lu S, Sheng D, Qian K, Cao J, Lu W, Zhang Q. A dual-ligand fusion peptide improves the brain-neuron targeting of nanocarriers in alzheimer’s disease mice. J Control Release. 2020;320:347–62. 31978446 10.1016/j.jconrel.2020.01.039

[181] Terashima T, Ogawa N, Nakae Y, Sato T, Katagi M, Okano J, Maegawa H, Kojima H. Gene therapy for neuropathic pain through siRNA-IRF5 gene delivery with homing peptides to microglia. Mol Ther Nucleic Acids. 2018;11:203–15. 29858055 10.1016/j.omtn.2018.02.007 PMC5992689

[182] Singh RSP, Dowty ME, Salganik M, Brodfuehrer JI, Walker GS, Sharma R, Beebe JS, Danto SI. A phase 1 study to assess mass balance and absolute bioavailability of Zimlovisertib in healthy male participants using a (14) C-Microtracer approach. Clin Pharmacol Drug Dev. 2022;11(7):815–25. 35506501 10.1002/cpdd.1109 PMC9322294

[183] Rao Q, Xu Y, Wang X, Luo H, Li H, Xiong J, Gao H, Cheng G. Construction of an intranasal drug delivery system with hypothalamus-targeting nanoparticles. SusMat. 2024;4(4):e222.

[184] Jin Z, Liu N, Wei H. Atherosclerosis is associated with amyloid and Tau pathology via blood-brain barrier dysfunction in the hippocampus of aged human brains. PLoS ONE. 2025;20(6):e0324652. 40498801 10.1371/journal.pone.0324652 PMC12157335

[185] Zhang Z, Jia G, Wang Q, Yu Y, Tang X, Zheng H, Yang X, Xiao Y, Ou Y, Jiang J, et al. Atherosclerosis enhances the efficacy of liposome-encapsulated Bromocriptine in reducing the incidence of prolactinemia in pituitary tumors. J Nanobiotechnol. 2025;23(1):392. 10.1186/s12951-025-03465-0 PMC12121278 40442804

[186] Obici S, Feng Z, Arduini A, Conti R, Rossetti L. Inhibition of hypothalamic carnitine palmitoyltransferase-1 decreases food intake and glucose production. Nat Med. 2003;9(6):756–61. 12754501 10.1038/nm873

[187] Rodriguez-Rodriguez R, Fosch A, Garcia-Chica J, Zagmutt S, Casals N. Targeting carnitine palmitoyltransferase 1 isoforms in the hypothalamus: A promising strategy to regulate energy balance. J Neuroendocrinol. 2023;35(9):e13234. 36735894 10.1111/jne.13234

[188] Zagmutt S, Mera P, Gonzalez-Garcia I, Ibeas K, Romero MDM, Obri A, Martin B, Esteve-Codina A, Soler-Vazquez MC, Bastias-Perez M, et al. CPT1A in AgRP neurons is required for sex-dependent regulation of feeding and thirst. Biol Sex Differ. 2023;14(1):14. 36966335 10.1186/s13293-023-00498-8 PMC10040140

[189] Loftus TM, Jaworsky DE, Frehywot GL, Townsend CA, Ronnett GV, Lane MD, Kuhajda FP. Reduced food intake and body weight in mice treated with fatty acid synthase inhibitors. Science. 2000;288(5475):2379–81. 10875926 10.1126/science.288.5475.2379

[190] Kumar MV, Shimokawa T, Nagy TR, Lane MD. Differential effects of a centrally acting fatty acid synthase inhibitor in lean and obese mice. Proc Natl Acad Sci U S A. 2002;99(4):1921–5. 11854492 10.1073/pnas.042683699 PMC122295

[191] Gao S, Lane MD. Effect of the anorectic fatty acid synthase inhibitor C75 on neuronal activity in the hypothalamus and brainstem. Proc Natl Acad Sci U S A. 2003;100(10):5628–33. 12724522 10.1073/pnas.1031698100 PMC156252

[192] Hu Z, Cha SH, Chohnan S, Lane MD. Hypothalamic malonyl-CoA as a mediator of feeding behavior. Proc Natl Acad Sci U S A. 2003;100(22):12624–9. 14532332 10.1073/pnas.1834402100 PMC240668

[193] Mera P, Bentebibel A, Lopez-Vinas E, Cordente AG, Gurunathan C, Sebastian D, Vazquez I, Herrero L, Ariza X, Gomez-Puertas P, et al. C75 is converted to C75-CoA in the hypothalamus, where it inhibits carnitine palmitoyltransferase 1 and decreases food intake and body weight. Biochem Pharmacol. 2009;77(6):1084–95. 19094968 10.1016/j.bcp.2008.11.020

[194] Garcia-Chica J, Paraiso WKD, Zagmutt S, Fosch A, Reguera AC, Alzina S, Sanchez-Garcia L, Fukushima S, Toh K, Casals N, et al. Nanomedicine targeting brain lipid metabolism as a feasible approach for controlling the energy balance. Biomater Sci. 2023;11(7):2336–47. 36804651 10.1039/d2bm01751b

[195] Dai Y, Wolfgang MJ, Cha SH, Lane MD. Localization and effect of ectopic expression of CPT1c in CNS feeding centers. Biochem Biophys Res Commun. 2007;359(3):469–74. 17559810 10.1016/j.bbrc.2007.05.161

[196] Rodriguez-Rodriguez R, Miralpeix C, Fosch A, Pozo M, Calderon-Dominguez M, Perpinya X, Vellvehi M, Lopez M, Herrero L, Serra D, et al. CPT1C in the ventromedial nucleus of the hypothalamus is necessary for brown fat thermogenesis activation in obesity. Mol Metab. 2019;19:75–85. 30448371 10.1016/j.molmet.2018.10.010 PMC6323189

[197] Pozo M, Rodriguez-Rodriguez R, Ramirez S, Seoane-Collazo P, Lopez M, Serra D, Herrero L, Casals N. Hypothalamic regulation of liver and muscle nutrient partitioning by Brain-Specific carnitine palmitoyltransferase 1 C in male mice. Endocrinology. 2017;158(7):2226–38. 28472467 10.1210/en.2017-00151

[198] Casals N, Zammit V, Herrero L, Fado R, Rodriguez-Rodriguez R, Serra D. Carnitine palmitoyltransferase 1 C: from cognition to cancer. Prog Lipid Res. 2016;61:134–48. 26708865 10.1016/j.plipres.2015.11.004

[199] Kim HI, Park J, Zhu Y, Wang X, Han Y, Zhang D. Recent advances in extracellular vesicles for therapeutic cargo delivery. Exp Mol Med. 2024;56(4):836–49. 38556545 10.1038/s12276-024-01201-6 PMC11059217

[200] Huang L, Wu E, Liao J, Wei Z, Wang J, Chen Z. Research advances of engineered exosomes as drug delivery carrier. ACS Omega. 2023;8(46):43374–87. 38027310 10.1021/acsomega.3c04479 PMC10666244

[201] Xu G, Jin J, Fu Z, Wang G, Lei X, Xu J, Wang J. Extracellular vesicle-based drug overview: research landscape, quality control and nonclinical evaluation strategies. Signal Transduct Target Ther. 2025;10(1):255. 40804047 10.1038/s41392-025-02312-w PMC12350758

[202] Zhang Y, Kim MS, Jia B, Yan J, Zuniga-Hertz JP, Han C, Cai D. Hypothalamic stem cells control ageing speed partly through Exosomal MiRNAs. Nature. 2017;548(7665):52–7. 28746310 10.1038/nature23282 PMC5999038

[203] Aakel N, Mohammed R, Fathima A, Kerzabi R, Abdallah A, Ibrahim WN. Role of exosome in solid cancer progression and its potential therapeutics in cancer treatment. Cancer Med. 2025;14(9):e70941. 40344389 10.1002/cam4.70941 PMC12063069

[204] Dai J, Su Y, Zhong S, Cong L, Liu B, Yang J, Tao Y, He Z, Chen C, Jiang Y. Exosomes: key players in cancer and potential therapeutic strategy. Signal Transduct Target Ther. 2020;5(1):145. 32759948 10.1038/s41392-020-00261-0 PMC7406508

[205] Kalluri R. The biology and function of extracellular vesicles in immune response and immunity. Immunity. 2024;57(8):1752–68. 39142276 10.1016/j.immuni.2024.07.009 PMC11401063

[206] Wang W, Qiao S, Kong X, Zhang G, Cai Z. The role of exosomes in immunopathology and potential therapeutic implications. Cell Mol Immunol. 2025;22(9):975–95. 40659888 10.1038/s41423-025-01323-5 PMC12398553

[207] He J, Ren W, Wang W, Han W, Jiang L, Zhang D, Guo M. Exosomal targeting and its potential clinical application. Drug Deliv Transl Res. 2022;12(10):2385–402. 34973131 10.1007/s13346-021-01087-1 PMC9458566

[208] Maas RR, Soukup K, Fournier N, Massara M, Galland S, Kornete M, Wischnewski V, Lourenco J, Croci D, Alvarez-Prado AF, et al. The local microenvironment drives activation of neutrophils in human brain tumors. Cell. 2023;186(21):4546–66. e4527. 37769657 10.1016/j.cell.2023.08.043

[209] Wang Y, Niu H, Li L, Han J, Liu Z, Chu M, Sha X, Zhao J. Anti-CHAC1 exosomes for nose-to-brain delivery of miR-760-3p in cerebral ischemia/reperfusion injury mice inhibiting neuron ferroptosis. J Nanobiotechnol. 2023;21(1):109. 10.1186/s12951-023-01862-x PMC10041751 36967397

[210] Cone AS, Yuan X, Sun L, Duke LC, Vreones MP, Carrier AN, Kenyon SM, Carver SR, Benthem SD, Stimmell AC, et al. Mesenchymal stem cell-derived extracellular vesicles ameliorate alzheimer’s disease-like phenotypes in a preclinical mouse model. Theranostics. 2021;11(17):8129–42. 34373732 10.7150/thno.62069 PMC8344012

[211] Delila L, Nebie O, Le NTN, Timmerman K, Lee DY, Wu YW, Chou ML, Buee L, Chou SY, Blum D, et al. Neuroprotective effects of intranasal extracellular vesicles from human platelet concentrates supernatants in traumatic brain injury and parkinson’s disease models. J Biomed Sci. 2024;31(1):87. 39237980 10.1186/s12929-024-01072-z PMC11375990

[212] Huang W, Zhang T, Li X, Gong L, Zhang Y, Luan C, Shan Q, Gu X, Zhao L. Intranasal administration of umbilical cord mesenchymal stem cell exosomes alleviates parkinson’s disease. Neuroscience. 2024;549:1–12. 38705349 10.1016/j.neuroscience.2024.04.010

[213] Martinez de Morentin PB, Urisarri A, Couce ML, Lopez M. Molecular mechanisms of appetite and obesity: a role for brain AMPK. Clin Sci (Lond). 2016;130(19):1697–709. 27555613 10.1042/CS20160048

[214] Le Stunff H, Brasilio Villalta P, Magnan C. Hypothalamic ceramide metabolism in obesity and dysregulation of glucose homeostasis. Rev Endocr Metab Disord. 2025. 10.1007/s11154-025-10003-8 41082169

[215] Alvarez-Crespo M, Csikasz RI, Martinez-Sanchez N, Dieguez C, Cannon B, Nedergaard J, Lopez M. Essential role of UCP1 modulating the central effects of thyroid hormones on energy balance. Mol Metab. 2016;5(4):271–82. 27069867 10.1016/j.molmet.2016.01.008 PMC4812006

[216] Capelli V, Grijota-Martinez C, Dragano NRV, Rial-Pensado E, Ferno J, Nogueiras R, Mittag J, Dieguez C, Lopez M. Orally induced hyperthyroidism regulates hypothalamic AMP-Activated protein kinase. Nutrients. 2021;13(12):4204. 34959756 10.3390/nu13124204 PMC8708331

[217] Lopez M, Varela L, Vazquez MJ, Rodriguez-Cuenca S, Gonzalez CR, Velagapudi VR, Morgan DA, Schoenmakers E, Agassandian K, Lage R, et al. Hypothalamic AMPK and fatty acid metabolism mediate thyroid regulation of energy balance. Nat Med. 2010;16(9):1001–8. 20802499 10.1038/nm.2207 PMC2935934

[218] Martinez-Sanchez N, Moreno-Navarrete JM, Contreras C, Rial-Pensado E, Ferno J, Nogueiras R, Dieguez C, Fernandez-Real JM, Lopez M. Thyroid hormones induce Browning of white fat. J Endocrinol. 2017;232(2):351–62. 27913573 10.1530/JOE-16-0425 PMC5292977

[219] Rial-Pensado E, Rivas-Limeres V, Grijota-Martinez C, Rodriguez-Diaz A, Capelli V, Barca-Mayo O, Nogueiras R, Mittag J, Dieguez C, Lopez M. Temperature modulates systemic and central actions of thyroid hormones on BAT thermogenesis. Front Physiol. 2022;13:1017381. 36467699 10.3389/fphys.2022.1017381 PMC9716276

[220] Lopez M, Tena-Sempere M. Estradiol effects on hypothalamic AMPK and BAT thermogenesis: A gateway for obesity treatment? Pharmacol Ther. 2017;178:109–22. 28351720 10.1016/j.pharmthera.2017.03.014

[221] Martinez de Morentin PB, Gonzalez-Garcia I, Martins L, Lage R, Fernandez-Mallo D, Martinez-Sanchez N, Ruiz-Pino F, Liu J, Morgan DA, Pinilla L, et al. Estradiol regulates brown adipose tissue thermogenesis via hypothalamic AMPK. Cell Metab. 2014;20(1):41–53. 24856932 10.1016/j.cmet.2014.03.031 PMC4082097

[222] Martinez de Morentin PB, Lage R, Gonzalez-Garcia I, Ruiz-Pino F, Martins L, Fernandez-Mallo D, Gallego R, Ferno J, Senaris R, Saha AK, et al. Pregnancy induces resistance to the anorectic effect of hypothalamic malonyl-CoA and the thermogenic effect of hypothalamic AMPK Inhibition in female rats. Endocrinology. 2015;156(3):947–60. 25535827 10.1210/en.2014-1611 PMC4330316

[223] Martins L, Seoane-Collazo P, Contreras C, Gonzalez-Garcia I, Martinez-Sanchez N, Gonzalez F, Zalvide J, Gallego R, Dieguez C, Nogueiras R, et al. A functional link between AMPK and orexin mediates the effect of BMP8B on energy balance. Cell Rep. 2016;16(8):2231–42. 27524625 10.1016/j.celrep.2016.07.045 PMC4999418

[224] Whittle AJ, Carobbio S, Martins L, Slawik M, Hondares E, Vazquez MJ, Morgan D, Csikasz RI, Gallego R, Rodriguez-Cuenca S, et al. BMP8B increases brown adipose tissue thermogenesis through both central and peripheral actions. Cell. 2012;149(4):871–85. 22579288 10.1016/j.cell.2012.02.066 PMC3383997

[225] Tanida M, Yamamoto N, Morgan DA, Kurata Y, Shibamoto T, Rahmouni K. Leptin receptor signaling in the hypothalamus regulates hepatic autonomic nerve activity via phosphatidylinositol 3-kinase and AMP-activated protein kinase. J Neurosci. 2015;35(2):474–84. 25589743 10.1523/JNEUROSCI.1828-14.2015 PMC4293404

[226] Tanida M, Yamamoto N, Shibamoto T, Rahmouni K. Involvement of hypothalamic AMP-activated protein kinase in leptin-induced sympathetic nerve activation. PLoS ONE. 2013;8(2):e56660. 23418591 10.1371/journal.pone.0056660 PMC3572050

[227] Beiroa D, Imbernon M, Gallego R, Senra A, Herranz D, Villarroya F, Serrano M, Ferno J, Salvador J, Escalada J, et al. GLP-1 agonism stimulates brown adipose tissue thermogenesis and Browning through hypothalamic AMPK. Diabetes. 2014;63(10):3346–58. 24917578 10.2337/db14-0302

[228] Martinez de Morentin PB, Whittle AJ, Ferno J, Nogueiras R, Dieguez C, Vidal-Puig A, Lopez M. Nicotine induces negative energy balance through hypothalamic AMP-activated protein kinase. Diabetes. 2012;61(4):807–17. 22315316 10.2337/db11-1079 PMC3314364

[229] Seoane-Collazo P, Dieguez C, Nogueiras R, Rahmouni K, Fernandez-Real JM, Lopez M. Nicotine’ actions on energy balance: friend or foe? Pharmacol Ther. 2021;219:107693. 32987056 10.1016/j.pharmthera.2020.107693

[230] Seoane-Collazo P, Martinez de Morentin PB, Ferno J, Dieguez C, Nogueiras R, Lopez M. Nicotine improves obesity and hepatic steatosis and ER stress in diet-induced obese male rats. Endocrinology. 2014;155(5):1679–89. 24517227 10.1210/en.2013-1839

[231] Seoane-Collazo P, Romero-Pico A, Rial-Pensado E, Linares-Pose L, Estevez-Salguero A, Ferno J, Nogueiras R, Dieguez C, Lopez M. kappa-Opioid signaling in the lateral hypothalamic area modulates Nicotine-Induced negative energy balance. Int J Mol Sci. 2021;22(4):1515. 33546289 10.3390/ijms22041515 PMC7913331

[232] Seoane-Collazo P, Roa J, Rial-Pensado E, Linares-Pose L, Beiroa D, Ruiz-Pino F, Lopez-Gonzalez T, Morgan DA, Pardavila JA, Sanchez-Tapia MJ, et al. SF1-Specific AMPKalpha1 deletion protects against Diet-Induced obesity. Diabetes. 2018;67(11):2213–26. 30104247 10.2337/db17-1538 PMC6198345

[233] Freire-Agulleiro O, Estevez-Salguero A, Ferreira V, Holleman CL, Garcia-Curras J, Gonzalez-Garcia I, Nogueiras R, Tena-Sempere M, Garcia-Caceres C, Dieguez C, et al. SF1-specific deletion of the energy sensor AMPKgamma2 induces obesity. Mol Metab. 2025;92:102091. 39746605 10.1016/j.molmet.2024.102091 PMC11782900

[234] Alvarez-Erviti L, Seow Y, Yin H, Betts C, Lakhal S, Wood MJ. Delivery of SiRNA to the mouse brain by systemic injection of targeted exosomes. Nat Biotechnol. 2011;29(4):341–5. 21423189 10.1038/nbt.1807

[235] Li X, Corbett AL, Taatizadeh E, Tasnim N, Little JP, Garnis C, Daugaard M, Guns E, Hoorfar M, Li ITS. Challenges and opportunities in exosome research-Perspectives from biology, engineering, and cancer therapy. APL Bioeng. 2019;3(1):011503. 31069333 10.1063/1.5087122 PMC6481742

[236] Yang M, Wu SY. The advances and challenges in utilizing exosomes for delivering cancer therapeutics. Front Pharmacol. 2018;9:735. 30061829 10.3389/fphar.2018.00735 PMC6055018

[237] Birch LA. Engineering extracellular vesicles for therapeutic delivery in ischaemic stroke. Nat Rev Cardiol. 2025;22(4):219. 39810049 10.1038/s41569-025-01122-5

[238] Manolopoulos A, Yao PJ, Kapogiannis D. Extracellular vesicles: translational research and applications in neurology. Nat Rev Neurol. 2025;21(5):265–82. 40181198 10.1038/s41582-025-01080-z

[239] Paganini C, Boyce H, Libort G, Arosio P. High-Yield production of extracellular vesicle subpopulations with constant quality using Batch-Refeed cultures. Adv Healthc Mater. 2023;12(8):e2202232. 36479632 10.1002/adhm.202202232 PMC11468747

[240] Xiao J, Sluijter JPG. Extracellular vesicles in cardiovascular homeostasis and disease: potential role in diagnosis and therapy. Nat Rev Cardiol. 2025;22(11):883–95. 40045042 10.1038/s41569-025-01141-2

[241] Wadenpohl T, Shein M, Steinberg J, Lehmann JB, Schütz AK, Jung S. Economical large-scale purification of extracellular vesicles from urine. Sep Purif Technol. 2024;335:126155.

[242] Palama MEF, Gorgun C, Rovere M, Shaw GM, Reverberi D, Formica M, Quarto E, Barry F, Murphy M, Gentili C. Batch variability and anti-inflammatory effects of iPSC-derived mesenchymal stromal cell extracellular vesicles in osteoarthritis in vitro model. Front Bioeng Biotechnol. 2025;13:1536843. 40242358 10.3389/fbioe.2025.1536843 PMC11999995

[243] Tertel T, Dittrich R, Arsene P, Jensen A, Giebel B. EV products obtained from iPSC-derived MSCs show batch-to-batch variations in their ability to modulate allogeneic immune responses in vitro. Front Cell Dev Biol. 2023;11:1282860. 37965578 10.3389/fcell.2023.1282860 PMC10642442

[244] Korchak JA, Wiest EF, Zubair AC. How do we assess batch-to-batch consistency between extracellular vesicle products? Transfusion 2023, 63(2):279–87. 36329638 10.1111/trf.17156

[245] Zhong J, Xia B, Shan S, Zheng A, Zhang S, Chen J, Liang XJ. High-quality milk exosomes as oral drug delivery system. Biomaterials. 2021;277:121126. 34544033 10.1016/j.biomaterials.2021.121126

[246] Munagala R, Aqil F, Jeyabalan J, Gupta RC. Bovine milk-derived exosomes for drug delivery. Cancer Lett. 2016;371(1):48–61. 26604130 10.1016/j.canlet.2015.10.020 PMC4706492

[247] Mierzejewska J, Kowalska P, Marlicka K, Dworakowska S, Sitkiewicz E, Trzaskowski M, Gluchowska A, Mosieniak G, Milner-Krawczyk M. Exploring extracellular vesicles of probiotic yeast as carriers of biologically active molecules transferred to human intestinal cells. Int J Mol Sci. 2023;24(14):11340. 37511103 10.3390/ijms241411340 PMC10379187

[248] Yuan M, Ma W, Liu B, Zou X, Huang B, Tian X, Jin Y, Zheng N, Wu Z, Wang Y. Delivery of therapeutic RNA by extracellular vesicles derived from Saccharomyces cerevisiae for medicine applications. J Pharm Sci. 2024;113(12):3574–85. 39454948 10.1016/j.xphs.2024.10.035

[249] Li T, Zhou Y, Wang H, Wang J, Lu R. Leveraging engineered yeast small extracellular vesicles serve as multifunctional platforms for effectively loading Methyl salicylate through the esterase-responsive active loading strategy. Eur J Pharm Biopharm. 2025;210:114696. 40113048 10.1016/j.ejpb.2025.114696

[250] Niu W, Xiao Q, Wang X, Zhu J, Li J, Liang X, Peng Y, Wu C, Lu R, Pan Y, et al. A biomimetic drug delivery system by integrating grapefruit extracellular vesicles and Doxorubicin-Loaded Heparin-Based nanoparticles for glioma therapy. Nano Lett. 2021;21(3):1484–92. 33475372 10.1021/acs.nanolett.0c04753

[251] Yazdanpanah S, Romano S, Valentino A, Galderisi U, Peluso G, Calarco A. Plant-derived exosomes: Carriers and cargo of natural bioactive compounds: emerging functions and applications in human health. Nanomaterials (Basel). 2025;15(13). 10.3390/nano15131005 PMC12251442 40648712

[252] Liu H, Dong T, Dong C, Yang F, Zhou Q, Guan C, Wang W. Plant-derived exosome-like nanovesicles: a novel therapeutic perspective for skin diseases. J Nanobiotechnol. 2025;23(1):640. 10.1186/s12951-025-03715-1 PMC12514849 41074041

[253] Wen Y, Fu Q, Soliwoda A, Zhang S, Zheng M, Mao W, Wan Y. Cell-derived nanovesicles prepared by membrane extrusion are good substitutes for natural extracellular vesicles. Extracell Vesicle. 2022;1:1004. 10.1016/j.vesic.2022.100004 PMC9794200 36578271

[254] Jang HJ, Shim KS, Lee J, Park JH, Kang SJ, Shin YM, Lee JB, Baek W, Yoon JK. Engineering of cell Derived-Nanovesicle as an alternative to exosome therapy. Tissue Eng Regen Med. 2024;21(1):1–19. 38066355 10.1007/s13770-023-00610-4 PMC10764700

[255] Yu Y, Cheng Q, Ji X, Chen H, Zeng W, Zeng X, Zhao Y, Mei L. Engineered drug-loaded cellular membrane nanovesicles for efficient treatment of postsurgical cancer recurrence and metastasis. Sci Adv. 2022;8(49):eadd3599. 36490349 10.1126/sciadv.add3599 PMC9733928

[256] Prasad R, Mendes BB, Gorain M, Chandra Kundu G, Gupta N, Peng B, Aung Win EH, Qing H, Conde J. Bioinspired and biomimetic cancer-cell-derived membrane nanovesicles for preclinical tumor-targeted nanotheranostics. Cell Rep Phys Sci. 2023;4(11):101648. 38021344 10.1016/j.xcrp.2023.101648 PMC10665589

[257] Soprano E, Polo E, Pelaz B, Del Pino P. Biomimetic cell-derived nanocarriers in cancer research. J Nanobiotechnol. 2022;20(1):538. 10.1186/s12951-022-01748-4 PMC9771790 36544135

[258] Zhong Z, Deng W, Wu J, Shang H, Tong Y, He Y, Huang Q, Ba X, Chen Z, Tang K. Cell membrane coated nanoparticles as a biomimetic drug delivery platform for enhancing cancer immunotherapy. Nanoscale. 2024;16(18):8708–38. 38634521 10.1039/d4nr00284a

[259] Gravan P, Marchal JA, Galisteo-Gonzalez F. Improving tumor treatment: cell membrane-coated nanoparticles for targeted therapies. Mater Today Bio. 2025;32:101716. 40391023 10.1016/j.mtbio.2025.101716 PMC12088825

[260] Li J, Wei Y, Zhang C, Bi R, Qiu Y, Li Y, Hu B. Cell-Membrane-Coated nanoparticles for targeted drug delivery to the brain for the treatment of neurological diseases. Pharmaceutics. 2023;15(2):621. 36839943 10.3390/pharmaceutics15020621 PMC9960717

[261] Goh WJ, Zou S, Ong WY, Torta F, Alexandra AF, Schiffelers RM, Storm G, Wang JW, Czarny B, Pastorin G. Bioinspired Cell-Derived nanovesicles versus exosomes as drug delivery systems: a Cost-Effective alternative. Sci Rep. 2017;7(1):14322. 29085024 10.1038/s41598-017-14725-x PMC5662560

[262] Soprano E, Migliavacca M, López-Ferreiro M, Pelaz B, Polo E, del Pino P. Fusogenic Cell-Derived nanocarriers for cytosolic delivery of cargo inside living cells. J Colloid Interface Sci. 2023;648:488–96. 37302232 10.1016/j.jcis.2023.06.015

[263] Migliavacca M, Correa-Paz C, Perez-Mato M, Bielawski PB, Zhang I, Marie P, Hervella P, Rubio M, Maysinger D, Vivien D, et al. Thrombolytic therapy based on lyophilized platelet-derived nanocarriers for ischemic stroke. J Nanobiotechnol. 2024;22(1):10. 10.1186/s12951-023-02206-5 PMC10763438 38166940

[264] Ou YH, Zou S, Goh WJ, Wang JW, Wacker M, Czarny B, Pastorin G. Cell-Derived nanovesicles as Exosome-Mimetics for drug delivery purposes: uses and recommendations. Methods Mol Biol. 2021;2211:147–70. 33336276 10.1007/978-1-0716-0943-9_11

[265] Jo W, Kim J, Yoon J, Jeong D, Cho S, Jeong H, Yoon YJ, Kim SC, Gho YS, Park J. Large-scale generation of cell-derived nanovesicles. Nanoscale. 2014;6(20):12056–64. 25189198 10.1039/c4nr02391a

[266] Picone P, Palumbo FS, Cancilla F, Girgenti A, Cancemi P, Muccilli V, Francesco AD, Cimino M, Cipollina C, Soligo M, et al. Brain biodistribution of Myelin nanovesicles with targeting potential for multiple sclerosis. Acta Biomater. 2024;187:352–65. 39159713 10.1016/j.actbio.2024.08.016

[267] Liu Z, Xia Q, Wang C, Xu J, Tian K, Wang Z, Li L, Li Y, Shang H, Liu Q, et al. Biomimetic astrocyte cell membrane-fused nanovesicles for protecting neurovascular units in hypoxic ischemic encephalopathy. J Nanobiotechnol. 2024;22(1):766. 10.1186/s12951-024-03053-8 PMC11656965 39695691

[268] Shen S, Cheng X, Zhou L, Zhao Y, Wang H, Zhang J, Sun X, Wang Y, Shu Y, Xu Y, et al. Neutrophil nanovesicle protects against experimental autoimmune encephalomyelitis through enhancing Myelin clearance by microglia. ACS Nano. 2022;16(11):18886–97. 36288210 10.1021/acsnano.2c07798

[269] Lin RR, Jin LL, Xue YY, Zhang ZS, Huang HF, Chen DF, Liu Q, Mao ZW, Wu ZY, Tao QQ. Hybrid Membrane-Coated nanoparticles for precise targeting and synergistic therapy in alzheimer’s disease. Adv Sci (Weinh). 2024;11(24):e2306675. 38647399 10.1002/advs.202306675 PMC11200089

[270] Sasannia S, Leigh R, Bastani PB, Shin HG, van Zijl P, Knutsson L, Nyquist P. Blood-brain barrier breakdown in brain ischemia: insights from MRI perfusion imaging. Neurotherapeutics. 2025;22(1):e00516. 39709246 10.1016/j.neurot.2024.e00516 PMC11840350

[271] Mathias K, Machado RS, Stork S, Dos Santos D, Joaquim L, Generoso J, Danielski LG, Barichello T, Prophiro JS, Petronilho F. Blood-brain barrier permeability in the ischemic stroke: an update. Microvasc Res. 2024;151:104621. 37918521 10.1016/j.mvr.2023.104621

[272] Li Q, Wang C, Hu J, Jiao W, Tang Z, Song X, Wu Y, Dai J, Gao P, Du L, et al. Cannabidiol-loaded biomimetic macrophage membrane vesicles against post-traumatic stress disorder assisted by ultrasound. Int J Pharm. 2023;637:122872. 36958611 10.1016/j.ijpharm.2023.122872

[273] Nance E, Timbie K, Miller GW, Song J, Louttit C, Klibanov AL, Shih TY, Swaminathan G, Tamargo RJ, Woodworth GF, et al. Non-invasive delivery of stealth, brain-penetrating nanoparticles across the blood-brain barrier using MRI-guided focused ultrasound. J Control Release. 2014;189:123–32. 24979210 10.1016/j.jconrel.2014.06.031 PMC4125545

[274] Fazal S, Lee R. Biomimetic bacterial membrane vesicles for drug delivery applications. Pharmaceutics. 2021;13(9):1430. 34575506 10.3390/pharmaceutics13091430 PMC8468068

[275] Qing G, Gong N, Chen X, Chen J, Zhang H, Wang Y, Wang R, Zhang S, Zhang Z, Zhao X, et al. Natural and engineered bacterial outer membrane vesicles. Biophys Rep. 2019;5(4):184–98.

[276] You H, Zhang S, Zhang Y, Chen Q, Wu Y, Zhou Z, Zhao Z, Su B, Li X, Guo Y, et al. Engineered bacterial outer membrane Vesicles-Based doxorubicin and CD47-siRNA Co-Delivery nanoplatform overcomes immune resistance to potentiate the immunotherapy of glioblastoma. Adv Mater. 2025;37(15):e2418053. 40035513 10.1002/adma.202418053

[277] Pascal TA, Abrol R, Mittal R, Wang Y, Prasadarao NV, Goddard WA 3. Experimental validation of the predicted binding site of Escherichia coli K1 outer membrane protein A to human brain microvascular endothelial cells: identification of critical mutations that prevent E. coli meningitis. J Biol Chem. 2010;285(48):37753–61. 20851887 10.1074/jbc.M110.122804 PMC2988380

[278] Pan J, Wang Z, Huang X, Xue J, Zhang S, Guo X, Zhou S. Bacteria-Derived Outer-Membrane vesicles hitchhike neutrophils to enhance ischemic stroke therapy. Adv Mater. 2023;35(38):e2301779. 37358255 10.1002/adma.202301779

[279] Tenchov R, Bird R, Curtze AE, Zhou Q. Lipid nanoparticles horizontal line from liposomes to mRNA vaccine Delivery, a landscape of research diversity and advancement. ACS Nano. 2021;15(11):16982–7015. 34181394 10.1021/acsnano.1c04996

[280] Xu S, Hu Z, Song F, Xu Y, Han X. Lipid nanoparticles: Composition, formulation, and application. Mol Ther Methods Clin Dev. 2025;33(2):101463. 40927763 10.1016/j.omtm.2025.101463 PMC12415982

[281] Wu S, Lin L, Shi L, Liu S. An overview of lipid constituents in lipid nanoparticle mRNA delivery systems. Wiley Interdiscip Rev Nanomed Nanobiotechnol. 2024;16(4):e1978. 38965928 10.1002/wnan.1978

[282] Hald Albertsen C, Kulkarni JA, Witzigmann D, Lind M, Petersson K, Simonsen JB. The role of lipid components in lipid nanoparticles for vaccines and gene therapy. Adv Drug Deliv Rev. 2022;188:114416. 35787388 10.1016/j.addr.2022.114416 PMC9250827

[283] Ma F, Yang L, Sun Z, Chen J, Rui X, Glass Z, Xu Q. Neurotransmitter-derived lipidoids (NT-lipidoids) for enhanced brain delivery through intravenous injection. Sci Adv. 2020;6(30):eabb4429. 32832671 10.1126/sciadv.abb4429 PMC7439549

[284] Pardi N, Tuyishime S, Muramatsu H, Kariko K, Mui BL, Tam YK, Madden TD, Hope MJ, Weissman D. Expression kinetics of nucleoside-modified mRNA delivered in lipid nanoparticles to mice by various routes. J Control Release. 2015;217:345–51. 26264835 10.1016/j.jconrel.2015.08.007 PMC4624045

[285] Tong H, Ma Z, Yu J, Li D, Zhu Q, Shi H, Wu Y, Yang H, Zheng Y, Sun D, et al. Optimizing Peptide-Conjugated lipid nanoparticles for efficient SiRNA delivery across the Blood-Brain barrier and treatment of glioblastoma multiforme. ACS Chem Biol. 2025;20(4):942–52. 40080657 10.1021/acschembio.5c00039

[286] Han EL, Tang S, Kim D, Murray AM, Swingle KL, Hamilton AG, Mrksich K, Padilla MS, Palanki R, Li JJ, et al. Peptide-Functionalized lipid nanoparticles for targeted systemic mRNA delivery to the brain. Nano Lett. 2025;25(2):800–10. 39688915 10.1021/acs.nanolett.4c05186

[287] Wang C, Xue Y, Markovic T, Li H, Wang S, Zhong Y, Du S, Zhang Y, Hou X, Yu Y, et al. Blood-brain-barrier-crossing lipid nanoparticles for mRNA delivery to the central nervous system. Nat Mater. 2025;24(10):1653–63. 39962245 10.1038/s41563-024-02114-5 PMC12514551

[288] Bian X, Guo Q, Yau LF, Yang L, Wang X, Zhao S, Wu S, Qin X, Jiang ZH, Li C. Berberine-inspired ionizable lipid for self-structure stabilization and brain targeting delivery of nucleic acid therapeutics. Nat Commun. 2025;16(1):2368. 40064874 10.1038/s41467-025-57488-0 PMC11893799

[289] Wu F, Li N, Xiao Y, Palanki R, Yamagata H, Mitchell MJ, Han X. Lipid nanoparticles for delivery of CRISPR gene editing components. Small Methods. 2025;10(2):e2401632. 40434188 10.1002/smtd.202401632 PMC12825352

[290] Chen K, Han H, Zhao S, Xu B, Yin B, Lawanprasert A, Trinidad M, Burgstone BW, Murthy N, Doudna JA. Lung and liver editing by lipid nanoparticle delivery of a stable CRISPR-Cas9 ribonucleoprotein. Nat Biotechnol. 2025;43(9):1445–57. 39415058 10.1038/s41587-024-02437-3 PMC12000389

[291] Han Z, Huang C, Luo T, Mirkin CA. A general genome editing strategy using CRISPR lipid nanoparticle spherical nucleic acids. Proc Natl Acad Sci U S A. 2025;122(36):e2426094122. 40906807 10.1073/pnas.2426094122 PMC12435252

[292] Rosenblum D, Gutkin A, Kedmi R, Ramishetti S, Veiga N, Jacobi AM, Schubert MS, Friedmann-Morvinski D, Cohen ZR, Behlke MA et al. CRISPR-Cas9 genome editing using targeted lipid nanoparticles for cancer therapy. Sci Adv. 2020;6(47). 10.1126/sciadv.abc9450 PMC7673804 33208369

